# Applications of Hydrogels for Next-Generation Batteries

**DOI:** 10.3390/gels11090757

**Published:** 2025-09-19

**Authors:** Sabuj Chandra Sutradhar, Nipa Banik, Md. Shahriar Ahmed, Hohyoun Jang, Kyung-Wan Nam, Mobinul Islam

**Affiliations:** 1 Department of Energy Materials Science and Engineering, Konkuk University, 268 Chungwon-aero, Chungju-si 27478, Republic of Korea; chandra@kku.ac.kr (S.C.S.); nipa1987@kku.ac.kr (N.B.);; 2 Department of Energy and Materials Engineering, Dongguk University-Seoul, Seoul 04620, Republic of Korea; shahriar.emcl@dgu.ac.kr (M.S.A.);

**Keywords:** hydrogels, synthesis methods, lithium-ion batteries, sodium-ion batteries, zinc-ion batteries, aluminum-ion batteries, magnesium-ion batteries

## Abstract

Hydrogels have garnered significant attention as multifunctional materials in next-generation rechargeable batteries due to their high ionic conductivity, mechanical flexibility, and structural tunability. This review presents a comprehensive overview of hydrogel types—including natural, synthetic, composite, carbon-based, conductive polymer, and MOF hydrogels—and their synthesis methods, such as chemical crosslinking, self-assembly, and irradiation-based techniques. Characterization tools like SEM, XRD, and FTIR are discussed to evaluate their microstructure and performance. In rechargeable batteries systems, hydrogels enhance ionic transport and mechanical stability, particularly in lithium-ion, sodium-ion, zinc-ion, magnesium-ion, and aluminum-ion batteries. Despite their advantages, hydrogels face challenges such as limited mechanical strength, reduced stability under extreme conditions, and scalability issues. Current research focuses on advanced formulations, self-healing mechanisms, and sustainable materials to overcome these limitations. This review highlights the pivotal role of hydrogels in shaping the future of flexible, high-performance, and environmentally friendly secondary batteries.

## 1. Introduction

The global energy landscape is undergoing a transformative shift driven by the urgent need to reduce carbon emissions, mitigate climate change, and transition toward sustainable energy systems. This transition demands the development of advanced materials and technologies capable of delivering high energy efficiency, long-term stability, and environmental compatibility. Energy storage and conversion devices—such as metal-ion batteries [1,2,3,4,5,6,7], supercapacitors [8,9,10], fuel cells [11,12], and solar cells [13]—are at the forefront of this revolution, serving as critical enablers for renewable energy integration, electric mobility, and smart grid systems [14,15].

Among these technologies, rechargeable batteries play a pivotal role. Their continued advancement depends on the development of innovative materials that can enhance energy density, charge transport efficiency, and device longevity [16]. In this context, hydrogels have emerged as a class of multifunctional materials with immense potential to overcome limitations in conventional battery components. Hydrogels are three-dimensional, hydrophilic polymer networks capable of retaining large amounts of water while maintaining structural integrity. Their unique combination of mechanical flexibility, high ionic conductivity, tunable porosity, and compatibility with diverse functional materials makes them ideal candidates for next-generation energy systems [17,18].

Recent research has demonstrated the versatility of hydrogels in various roles across energy devices. In batteries, hydrogels have been employed as solid-state electrolytes, separators, and electrode binders, offering improved ion transport, enhanced interfacial contact, and mechanical adaptability [19,20]. Moreover, hydrogels have being explored as electrolyte matrices for energy storage materials [21,22]. The development of multifunctional hydrogels—engineered to exhibit properties such as self-healing [23], stretchability [24], thermo responsiveness [25], and shape memory [26]—has further expanded their applicability. These advanced hydrogels can adapt to mechanical deformation, recover from damage, and respond to environmental stimuli, thereby enhancing the durability and reliability of energy devices [27,28]. For instance, self-healing hydrogels can autonomously repair microcracks in electrodes or electrolytes, extending device lifespan [29,30], while stretchable hydrogels enable the fabrication of flexible and wearable energy systems, aligning with the growing demand for portable and body-integrated electronics [31].

Hydrogels have shown advanced suitability across multiple components of battery systems, particularly as electrolytes, electrode binders, and separators [32,33,34,35,36,37,38]. As electrolytes, hydrogels offer high ionic conductivity and enhanced safety by eliminating flammable solvents, making them superior to traditional liquid electrolytes [39,40,41]. Their soft, hydrated networks facilitate efficient ion transport while suppressing dendrite formation and side reactions [42,43,44,45]. As binders and separators, hydrogels improve interfacial contact between electrodes and electrolytes, accommodate volume changes during cycling, and maintain mechanical integrity under deformation [46,47,48,49,50]. Moreover, multifunctional hydrogels—engineered with properties such as self-healing, stretchability, and stimuli-responsiveness—enable the development of flexible, wearable, and bio-integrated energy devices, which are difficult to achieve with conventional solid-state or polymeric materials [51,52,53,54,55]. These advantages position hydrogels as transformative materials for next-generation battery architectures.

The rapid evolution of energy storage technologies has driven the search for safer, more flexible, and environmentally sustainable battery systems. Among emerging materials, hydrogels stand out for their ability to bridge the gap between liquid electrolyte-based and solid-state battery systems. Traditional liquid electrolytes offer high ionic conductivity but suffer from flammability, leakage, and dendrite formation. As reviewed by M. Li et al. [56], these systems also face challenges in long-term stability and compatibility with flexible or wearable devices.

Conversely, all-solid-state batteries (ASSBs) provide improved safety and energy density but are limited by poor interfacial contact and low room temperature ionic conductivity. Surendran et al. [57] highlight critical issues such as stack pressure and solid–solid interface degradation, which hinder scalability and reliability. In contrast, hydrogel-based batteries offer a hybrid solution—combining the mechanical integrity and safety of solid-state systems with the ionic transport efficiency of liquid electrolytes. Recent advances, as reported in Advanced Functional Materials [58], demonstrate how engineered hydrogel electrolytes can suppress side reactions, extend voltage windows, and maintain volume retention over extended cycles. These systems also enable interface engineering, self-healing, and biocompatibility, making them ideal for wearable electronics, bio-integrated devices, and flexible energy storage platforms.

Despite these promising attributes, several challenges hinder the widespread adoption of hydrogel-based materials in commercial energy technologies [59]. Key issues include long-term stability under fluctuating environmental conditions, scalability of synthesis, and integration with existing device architectures [32,59,60,61,62]. Addressing these challenges requires interdisciplinary approaches that combine polymer chemistry, nanotechnology, electrochemistry, and device engineering. Strategies such as nanoparticle reinforcement, dual-network structuring, and hybridization with conductive polymers or carbon-based materials are being actively explored to enhance the mechanical and electrochemical performance of hydrogels [63,64,65,66].

Given the growing interest in hydrogel-based materials for battery applications, a systematic and up-to-date review is essential to consolidate recent findings and provide clarity on emerging trends. While many studies have examined the electrochemical and mechanical properties of hydrogels, the rapid development of multifunctional designs—such as self-healing, stretchable, and stimuli-responsive systems—necessitates a thorough reassessment of their roles in energy storage and conversion.

This review aims to provide a comprehensive overview of recent advances in hydrogel materials for battery materials. We discuss the fundamental properties that make hydrogels suitable for battery applications, their synthesis and functionalization strategies, and their roles in various energy systems. Furthermore, we highlight current limitations and outline future research directions, emphasizing the potential of multifunctional hydrogels to contribute meaningfully to the development of sustainable, high-performance energy technologies.

## 2. Hydrogel Fundamentals for Battery Applications

Hydrogels are soft, water-rich polymer networks that have emerged as promising materials for energy storage systems due to their unique combination of ionic conductivity, mechanical flexibility, and chemical tunability. Their ability to retain large volumes of aqueous electrolytes while maintaining structural integrity makes them ideal for use in quasi-solid-state, flexible, and wearable batteries. This section introduces the foundational science behind hydrogels and explains how their internal structure and crosslinking mechanisms influence their performance in battery applications.

### 2.1. Matrix Structures and Crosslinking Mechanisms

The performance of hydrogels in battery systems is largely determined by their internal matrix structure and the nature of the crosslinking that stabilizes the polymer network. Crosslinking affects not only the mechanical strength and elasticity of the hydrogel but also its porosity, swelling behavior, and ion transport capabilities.

**Scheme 1:**
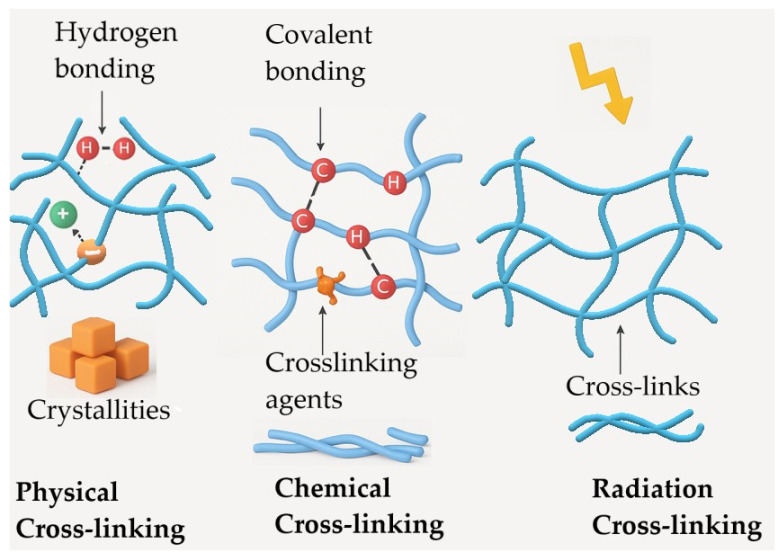
Overview of common Hydrogel Preparation Techniques.

Hydrogels are typically synthesized via three principal approaches: physical crosslinking, chemical crosslinking, and irradiation-based crosslinking, each producing networks with distinct characteristics. These methods differ in the type of cross-links formed, the stability of the resulting hydrogel, and the complexity of synthesis (Scheme 1).

#### 2.1.1. Physical CrossLinking

**Scheme 2:**
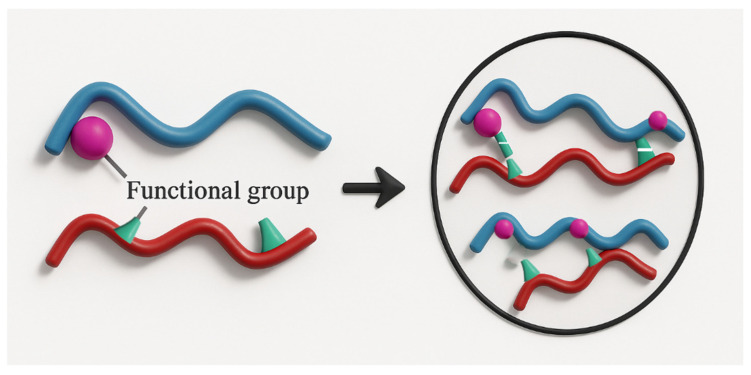
Schematic illustration of hydrogel synthesis using physical crosslinking method. Adapted and redrawn with permission from [70], Springer Nature 2021.

Physical crosslinking is widely favored for its simplicity and the absence of chemical crosslinkers, which enhances biocompatibility [67]. This method relies on non-covalent interactions such as hydrogen bonding, ionic interactions, and hydrophobic forces, resulting in reversible gel formation [68,69]. Several techniques are employed to achieve physical crosslinking (Scheme 2), including [70]

- Thermal cycling (heating or cooling of polymer solutions);
- Ionic interactions between charged polymer segments;
- Complex coacervation, involving phase separation of oppositely charged polymers;
- Hydrogen bonding between functional groups;
- Maturation, or heat-induced aggregation;
- Freeze–thaw cycles, which promote crystallite formation and physical entanglement.

Physically crosslinked hydrogels differ significantly from covalently bonded systems in their swelling behavior. Osmotic stress drives the rearrangement of polymer chains, allowing the hydrogel to expand while maintaining a stable network structure [70]. These hydrogels are particularly attractive for biomedical and sensor applications due to their uniform thermal properties and ease of fabrication.

Their inherent biocompatibility makes them suitable for tissue engineering and regenerative medicine. For example, physically crosslinked hyaluronic acid hydrogels have demonstrated enhanced mechanical properties and controlled biodegradation, supporting their use in injectable scaffolds, drug delivery platforms, and wound healing therapies [71].

#### 2.1.2. Chemical CrossLinking

Chemical crosslinking involves the formation of covalent bonds between polymer chains, either through the incorporation of reactive monomers or the use of crosslinking agents [72,73]. This method enables the synthesis of robust hydrogel networks with tailored mechanical and functional properties. Common crosslinkers include Formaldehyde [74], Glutaraldehyde [75], Oxidized dextrins [76], Oxidized alginate [77], Borax [78].

**Scheme 3:**
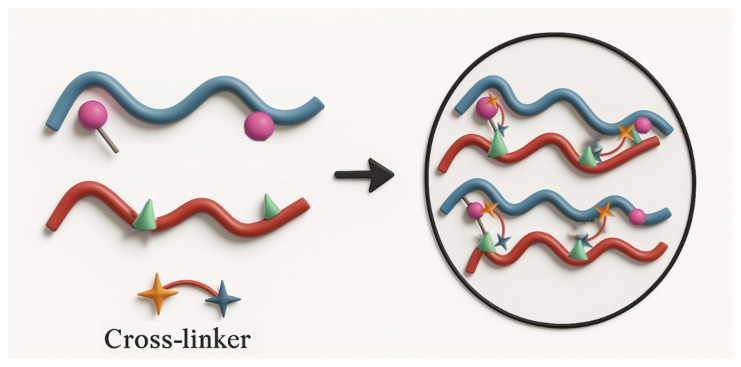
Schematic illustration of hydrogel synthesis using the chemical crosslinking method. Adapted and redrawn with permission from [70], Springer Nature 2021.

Two primary strategies are used in chemical crosslinking: blending, where polymers are physically mixed and then crosslinked, and grafting, where functional groups are covalently attached to polymer backbones [70,79] (Scheme 3).

Chemical crosslinking can be enhanced using UV irradiation, which facilitates the formation of biopolyesters with applications in medical and industrial fields. This method yields permanent hydrogels due to the covalent nature of the bonds formed, often involving small molecule crosslinkers, polymer–polymer conjugation, photosensitive agents, or enzyme-mediated reactions [79].

Hydrogels synthesized via chemical crosslinking—such as gelatin methacrylate and acrylic acid-based systems—are widely used in tissue regeneration (e.g., skin, bone, cartilage), microfluidic device fabrication, cell culture studies, biosensing, and drug/gene delivery due to their improved mechanical strength and biocompatibility.

#### 2.1.3. Radiation Crosslinking

**Scheme 4:**
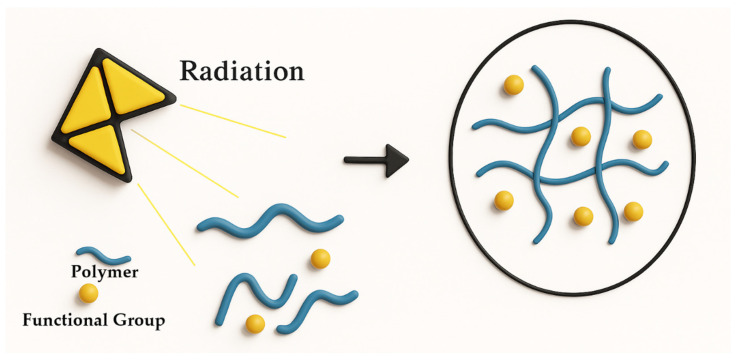
Schematic illustration of hydrogel synthesis using the radiation crosslinking method. Adapted and redrawn with permission from [70], Springer Nature 2021.

Radiation-induced crosslinking is a clean and efficient method that preserves the biocompatibility of polymers by avoiding chemical additives [80]. This technique involves the generation of free radicals within the polymer matrix upon exposure to high-energy radiation sources such as gamma rays, electron beams, or X-rays [81,82]. The effectiveness of radiation crosslinking depends on the polymer’s physical state and the surrounding environment. It can be performed in aqueous solutions, pastes, or solid-state formats [70,82] (Scheme 4).

Hydrogels produced through radiation crosslinking exhibit enhanced gel strength and elongation properties, making them suitable for demanding applications [83]. Ionizing radiation has also been used to modify synthetic polymers, improving their swelling behavior and mechanical performance. These hydrogels often respond to temperature changes, classifying them as stimuli-responsive materials [84,85].

For instance, polyvinylpyrrolidone (PVP) hydrogels formed via gamma or electron beam irradiation have shown promise in wound dressing applications due to their excellent biocompatibility, environmental stability, and ability to maintain moisture balance. Their potential extends to prosthetics and controlled drug delivery systems.

Various strategies have been employed for hydrogel synthesis, broadly categorized into physical, chemical, and irradiation-based crosslinking methods. Each method offers distinct advantages in terms of mechanical strength, biocompatibility, and functional tunability. The choice of crosslinking approach and formulation parameters—such as polymer concentration, pH, temperature, and crosslinker type—significantly influences the structural and functional properties of the resulting hydrogels. Table 1 summarizes representative examples from the literature, highlighting the materials used, synthesis conditions, and key characterization techniques. This comprehensive overview provides insight into the design flexibility and application potential of hydrogel systems.

**Table 1:** Representative hydrogel systems categorized by crosslinking method, synthesis parameters, and characterization techniques.

Crosslinking Method	Hydrogel Composition	Key Synthesis Parameters	Characterization Techniques	Ref.
Physical Crosslinking	Chitosan–Silver Nanocomposite	Polymer ratio, pH, ionic strength	Sol–gel transition, swelling behavior, antimicrobial activity, mechanical strength, SEM analysis	[86]
	Catechol–Chitosan–Thiolated Pluronic F-127	Polymer concentration, temperature	Tissue adhesion, mechanical stability, rheology	[87]
	Chitosan-based Hydrogel	pH, concentration, temperature	FTIR, rheological properties, mechanical testing, degradation profile	[88]
Chemical Crosslinking	Polyvinyl Alcohol/Carbomer/Glabridin	Crosslinker concentration, mixing time	Swelling ratio, mechanical strength, shape retention	[89]
	TiO_2_–Chitosan–Poly(acrylic acid)	Nanoparticle loading, pH	Dye adsorption, FTIR, SEM, thermal analysis	[90]
	Polyethylene Glycol Hydrogel	Mixing duration, temperature	Rheology, mechanical testing, swelling kinetics	[91]
	Acylated Xylan–Graphene Oxide	pH, polymer ratio	FTIR, SEM, particle size distribution	[91]
	Chitosan/Dopamine/Inulin Aldehyde	Composition ratio, reaction time	FTIR, DSC, mechanical testing, cytotoxicity assays	[92]
	Polyvinyl Alcohol Hydrogel	Polymer concentration, crosslinking agent	Tensile strength, rheology, SEM, FTIR, structural analysis	[93]
Irradiation Crosslinking	Chitosan–Silver Nanoparticles	Radiation dose, composition	SEM, UV–Vis absorbance, particle size, antimicrobial testing	[94]
	Sterculia Gum–Graphene Oxide	Radiation intensity, polymer ratio	SEM, swelling behavior, drug release profile, and biomedical evaluation	[95]

### 2.2. Organic Functional Groups Relevant to Battery Chemistry

Organic functional groups embedded within hydrogel networks critically influence their physicochemical properties and electrochemical performance in battery systems. Groups such as hydroxyl (-OH), carboxyl (-COOH), amine (-NH_2_), and sulfonic acid (-SO_3_H) enhance water retention, ion transport, and compatibility with electrode materials. Hydroxyl groups, prevalent in cellulose and poly(vinyl alcohol), facilitate hydrogen bonding and Zn^2^⁺ ion anchoring, reducing dendrite formation [96,97]. Carboxyl groups in poly(acrylic acid) and alginate coordinate multivalent cations and expand hydrogel porosity via electrostatic repulsion [98]. Amine groups in chitosan and PEI contribute to metal-ion coordination and proton conduction, with quaternary ammonium modifications further boosting conductivity [99]. Sulfonic acid groups, found in sulfonated polymers, offer high proton conductivity and stabilize the solid–electrolyte interphase (SEI) [32]. Synergistic designs combining multiple functional groups enable tailored ionic pathways, mechanical resilience, and electrochemical stability, vital for flexible and biocompatible energy storage applications [100].

### 2.3. Interfacial Traits and Ion Transport Mechanisms

The interface between hydrogel electrolytes and battery electrodes plays a pivotal role in determining electrochemical performance, where hydrogels act as soft, hydrated interlayers that facilitate ion exchange and stabilize interfacial dynamics. Their porous architecture supports continuous ion-conducting pathways filled with aqueous electrolytes, enabling localized ion transport via interactions with functional groups like –COOH and –SO_3_H [101]. This structure enhances ionic conductivity and suppresses dendrite growth, as seen in cellulose-based hydrogels anchoring Zn^2^⁺ ions [102]. The conformal nature of hydrogels reduces interfacial resistance and prevents mechanical degradation, while their ion reservoir capability ensures uniform ion flux and mitigates dendrite formation [103]. Functional groups such as carboxyl, sulfonic acid, and amine coordinate metal ions and guide ion migration through electrostatic interactions, improving selectivity and transport efficiency, especially in multivalent systems. Incorporation of conductive nanomaterials like CNTs, GO, and metal nanoparticles further enhances electron transport and mechanical resilience, forming percolation networks that support high-rate battery operation. CNT-reinforced hydrogels, for instance, demonstrate superior conductivity and flexibility, enabling stable performance under mechanical stress [104]. These interfacial traits make hydrogels ideal for next-generation energy storage systems, particularly in flexible and wearable applications.

### 2.4. Mechanical Properties and Durability Factors

In electrochemical energy storage systems, hydrogels serve as dynamic interfacial layers that enhance ion transport and stability between electrodes and electrolytes due to their soft, hydrated, and porous nature. Their polymeric matrix, enriched with functional groups like –COOH, –SO_3_H, and –OH, enables localized ion transport by forming coordination sites that improve selectivity and conductivity, as demonstrated in cellulose-based hydrogels anchoring Zn^2^⁺ ions and sulfonated hydrogels facilitating proton conduction [105]. Hydrogels also suppress dendrite formation by maintaining intimate contact with electrode surfaces, reducing interfacial resistance, and uniformly distributing metal ions to minimize concentration gradients and mechanical stress [106]. To further boost electrochemical performance, conductive nanomaterials such as CNTs, GO, and metal nanoparticles are integrated into hydrogels, forming percolation networks that enhance electron transfer and mechanical resilience [107]. CNT-reinforced hydrogels, for instance, exhibit superior conductivity and flexibility, supporting stable operation in stretchable battery systems. These interfacial traits make hydrogels a versatile platform for designing safe, high-performance, and mechanically adaptive batteries suitable for wearable and flexible electronics.

## 3. Classification of Hydrogels Used in Battery Systems

**Scheme 5:**
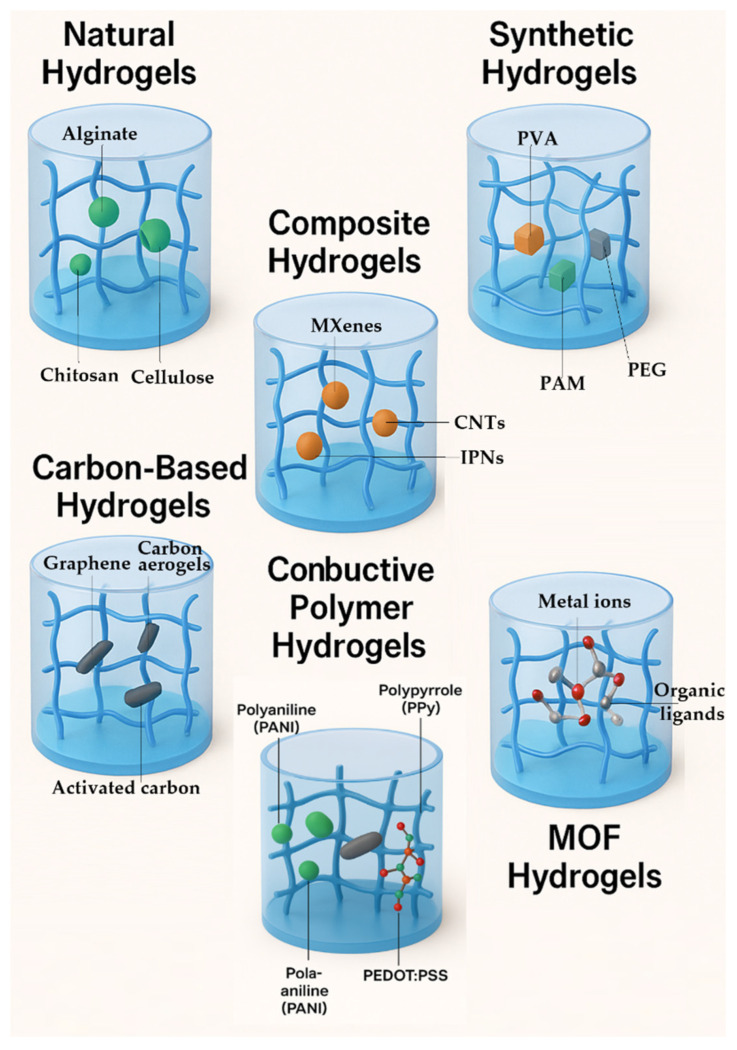
Schematic illustration of the types of hydrogels used in energy applications.

Hydrogels, with their three-dimensional polymeric networks and high water content, have become indispensable materials in energy storage and conversion technologies. Their ability to facilitate ion transport, maintain mechanical flexibility, and incorporate functional additives makes them ideal for use in batteries, supercapacitors, fuel cells, solar cells, and metal-air batteries. Based on their origin, composition, and functionalization, hydrogels used in energy applications can be broadly categorized into six main types: natural, synthetic, composite, carbon-based, conductive polymer, and metal–organic framework (MOF) hydrogels (Scheme 5).

### 3.1. Natural Hydrogels

Natural hydrogels, derived from renewable biopolymers such as alginate, chitosan, cellulose, starch, and gelatin, are increasingly recognized for their potential in sustainable energy technologies due to their biodegradability, biocompatibility, and environmental friendliness [108]. These hydrogels are composed of three-dimensional polymeric networks formed through physical crosslinking (e.g., hydrogen bonding, ionic interactions) or chemical crosslinking (e.g., covalent bonding via agents like glutaraldehyde or genipin) [109,110]. Their structural integrity and performance are largely governed by the presence of hydrophilic functional groups—such as carboxylate (–COO^−^), hydroxyl (–OH), and amino (–NH_2_)—which facilitate water retention, ion transport, and electrolyte compatibility [111]. For instance, alginate hydrogels, rich in carboxylate groups, form ionically crosslinked networks with divalent cations like Ca^2^⁺, creating porous matrices that enable efficient Zn^2^⁺ transport and suppress dendrite formation in zinc-ion batteries, as demonstrated in recent studies reporting ionic conductivities around 10^−3^ S/cm [112]. Chitosan hydrogels, containing amino and hydroxyl groups, exhibit proton conductivity and have been employed as proton-conducting separators in supercapacitors, where their protonated amino groups enhance ionic mobility under acidic conditions [113]. Similarly, cellulose-based hydrogels, particularly carboxymethyl cellulose (CMC), utilize hydroxyl and carboxyl groups to improve adhesion, ion diffusion, and mechanical stability when used as binders and separators in lithium-ion batteries [114]. Despite their advantages in sustainability and ionic conductivity, natural hydrogels face challenges such as low mechanical strength, limited electrochemical stability in high-voltage systems, and susceptibility to microbial degradation [115]. To address these limitations, recent innovations have focused on hybridizing natural hydrogels with nanomaterials or conductive polymers—for example, alginate–MXene composites have shown enhanced conductivity and mechanical resilience in flexible supercapacitors [116], chitosan–graphene oxide hydrogels have improved electron transfer in biofuel cells [117], and nanocellulose-based hydrogels functionalized with chiral biomolecules are being explored for multifunctional energy and biomedical applications [117,118,119,120].

### 3.2. Synthetic Hydrogels

Synthetic hydrogels, primarily based on polymers such as polyacrylamide (PAM), polyvinyl alcohol (PVA), polyethylene glycol (PEG), and polyacrylic acid (PAA), offer precise control over chemical composition, crosslinking density, and network architecture, making them highly tunable for energy applications [121,122,123]. These hydrogels are typically formed through chemical crosslinking (e.g., covalent bonding via crosslinkers or initiators) or physical crosslinking (e.g., hydrogen bonding, crystallization, or freeze–thaw cycles), resulting in robust three-dimensional networks with adjustable porosity and mechanical strength [123,124,125]. The presence of hydrophilic functional groups such as –OH, –COOH, and –CONH_2_ facilitates water absorption, ion transport, and compatibility with electrolytes [126]. For instance, PAM-based hydrogels, known for their flexibility and hydrogen-bonded networks, have been used as quasi-solid-state electrolytes in zinc-ion batteries, where they suppress dendrite formation and maintain ionic conductivity under mechanical deformation [33]. Similarly, PVA hydrogels, often reinforced with polyhydric additives or blended with cellulose, exhibit excellent thermal stability and wide-temperature adaptability (−70 to 100 °C), making them suitable for wearable and harsh-environment energy devices [127]. PEG and PEGDA-based hydrogels, due to their biocompatibility and tunable crosslinking, have been employed in flexible supercapacitors and lithium-ion batteries, where they serve as ion-conductive matrices with good electrochemical stability [128,129]. These synthetic hydrogels can also be engineered into dual-network or interpenetrating polymer networks (IPNs) to enhance mechanical resilience and self-healing properties [130]. Despite their versatility, challenges remain, including synthesis complexity, environmental persistence, and limited biodegradability. To address these, recent innovations have focused on integrating synthetic hydrogels with nanomaterials or conductive polymers to form multifunctional composites with enhanced conductivity, stretchability, and durability for next-generation energy storage and wearable electronics [63,65,131].

### 3.3. Composite Hydrogels

Composite hydrogels are multifunctional materials formed by integrating nanomaterials—such as carbon nanotubes (CNTs), graphene, MXenes, metal nanoparticles, or liquid metals—into hydrogel matrices, resulting in synergistic enhancements in electrical conductivity, mechanical strength, and functional responsiveness [63,132,133]. Structurally, these hydrogels consist of interpenetrating polymer networks (IPNs) or dual-network architectures, where the polymer chains are chemically or physically crosslinked, and the nanomaterials are either embedded within the network or adsorbed onto the polymer backbone [63,134,135]. This configuration enables the formation of continuous conductive pathways while maintaining the hydrogel’s inherent flexibility and water retention. For instance, MXene-based composite hydrogels have demonstrated high stretchability, conductivity, and self-healing properties, making them ideal for wearable sensors and energy devices [136,137]. Carbon nanomaterial-enhanced hydrogels, such as those incorporating graphene or CNTs, have been used to fabricate flexible pressure sensors and supercapacitor electrodes, where their high surface area and conductivity support rapid charge transport and mechanical adaptability [138,139]. Metal nanoparticle-infused hydrogels, including those with nanoscale liquid metals, offer exceptional plasticity and conductivity, enabling applications in electronic skin and soft robotics [140,141]. These composite systems often exploit triboelectric nanogenerator (TENG) mechanisms, stress-resistance responses, or electrophysiological acquisition, allowing for self-powered sensing and real-time physiological monitoring [142,143]. The incorporation of nanomaterials also enhances the hydrogel’s self-healing ability, achieved through reversible interactions such as hydrogen bonding, metal-ligand coordination, or dynamic covalent bonding [144,145]. Despite their promising performance, challenges remain in achieving uniform dispersion of nanomaterials, maintaining long-term stability, and scaling up fabrication. Nevertheless, composite hydrogels represent a transformative class of materials for next-generation energy storage, wearable electronics, and human–machine interface technologies [146,147,148,149].

### 3.4. Carbon-Based Hydrogels

Carbon-based hydrogels are a class of multifunctional materials that integrate carbon-rich components—such as graphene, carbon nanotubes (CNTs), activated carbon, carbon aerogels, and biomass-derived carbon—into hydrogel networks to enhance their electrical conductivity, mechanical strength, and electrochemical performance [150,151,152,153]. Structurally, these hydrogels consist of three-dimensional porous polymeric networks that are either chemically or physically crosslinked, with carbon materials embedded or interwoven within the matrix. The carbon fillers contribute to the formation of continuous electron-conducting pathways, while the hydrogel matrix retains water and facilitates ion transport [154]. For example, bilayer carbon-based hydrogel composites have demonstrated exceptional solar energy conversion efficiency (up to 93.7%) and high evaporation rates under simulated sunlight, owing to their hierarchical structure and photothermal properties [155]. Carbon aerogels, synthesized via sol–gel processes followed by freeze-drying and pyrolysis, offer high surface area, tunable pore size, and excellent conductivity, making them ideal for supercapacitor electrodes and battery components [156]. These materials often exhibit hierarchical porosity, which enhances electrolyte accessibility and charge storage capacity. Additionally, biomass-derived carbon hydrogels, fabricated from natural precursors like cellulose or lignin, provide sustainable alternatives with customizable surface chemistry and electrochemical behavior [112]. The incorporation of carbon nanostructures also improves the hydrogel’s mechanical resilience, enabling stretchability, compressibility, and self-healing properties [157,158]. However, challenges such as filler aggregation, interfacial compatibility, and long-term stability remain critical for practical deployment [159]. Recent innovations have focused on bilayer architectures, gradient structures, and hybrid composites to optimize performance across diverse energy applications, including supercapacitors, metal–air batteries, solar evaporators, and wearable electronics [140,160,161]. 

### 3.5. Conductive Polymer Hydrogels

Conductive polymer hydrogels (CPHs) are a unique class of hydrogels that combine the hydrophilic, stretchable nature of polymer networks with the electrical conductivity of intrinsically conductive polymers such as polyaniline (PANI), polypyrrole (PPy), and poly(3,4-ethylenedioxythiophene): polystyrene sulfonate (PEDOT:PSS) [64,162,163,164]. Structurally, CPHs are composed of three-dimensional crosslinked polymer networks—formed via chemical or physical crosslinking—into which conductive polymers are either embedded or polymerized in situ [27,165]. These networks often feature π-conjugated systems that facilitate electron delocalization and charge transport, while hydrophilic groups such as –SO_3_H, –NH_2_, and –OH enable water retention and ionic mobility [166,167]. For example, a γ-PGA/PEDOT:PSS hydrogel was synthesized using in situ polymerization, where the PEDOT:PSS content was tuned to modulate pore size, electromechanical properties, and swelling behavior, resulting in enhanced capacitance and mechanical performance for wearable sensors [168]. Similarly, polyaniline particles embedded in a PEDOT matrix have demonstrated rapid electron transfer and high capacitance, making them suitable for supercapacitor electrodes [169]. These hydrogels can be engineered into single-network, double-network, or multi-network architectures, often incorporating additional conductive fillers such as carbon nanotubes or metal nanowires to further improve conductivity and mechanical resilience [170]. CPHs exhibit multifunctional properties including self-healing, stretchability, anti-freezing, and stimuli-responsiveness, which are critical for applications in flexible electronics, electronic skin, bioelectronic interfaces, and energy storage devices [167,171]. However, challenges such as synthesis complexity, potential cytotoxicity of monomers, and long-term durability under cyclic mechanical stress remain [28,172,173] Recent innovations have focused on freeze-polymerization techniques, dopant engineering, and hybrid composite designs to overcome these limitations and expand the applicability of CPHs in next-generation wearable and implantable energy systems [28,174,175,176].

### 3.6. MOF Hydrogels

Metal–organic framework (MOF) hydrogels represent a novel class of hybrid materials that combine the porous crystalline architecture of MOFs with the hydrated, flexible polymeric networks of hydrogels, offering synergistic properties for advanced energy applications [177,178,179]. Structurally, MOFs are composed of metal ions or clusters coordinated with organic ligands, forming highly ordered frameworks with tunable pore sizes, high surface area, and customizable chemical functionality [180,181]. When integrated into hydrogel matrices—either by physical embedding, covalent bonding, or in situ growth—these MOFs enhance the hydrogel’s ion transport, mechanical strength, and electrochemical activity [182,183]. The resulting MOF hydrogels often exhibit interpenetrating networks, where the hydrogel provides water retention and flexibility, while the MOF domains contribute to selective ion adsorption, catalytic activity, and charge storage. Recent studies have demonstrated the use of MOF hydrogels in aqueous zinc-ion batteries, alkali-metal-ion batteries, and supercapacitors, where they serve as electrolytes, electrode scaffolds, or separator membranes, helping to mitigate dendrite growth and shuttle effects [179,184]. For example, ZIF-8 and MIL-101-based hydrogels have shown enhanced ionic conductivity and stability under cycling conditions, owing to their hierarchical porosity and functionalized surfaces [185,186]. Moreover, the incorporation of MOFs into hydrogels improves mechanical resilience, overcoming the brittleness of standalone MOFs and enabling stretchable or wearable device configurations [144,187]. Despite these advantages, challenges remain in terms of MOF dispersion, interfacial compatibility, and scalability of synthesis [183]. To address these, researchers are exploring mixed-ligand strategies, post-synthetic modifications, and composite designs that integrate MOFs with conductive polymers or carbon nanomaterials. These innovations are paving the way for MOF hydrogels to be used not only in energy storage but also in electrocatalysis, water purification, and bioelectronic systems, where multifunctionality and structural tunability are essential [188].

Each type of hydrogel offers unique advantages and faces specific challenges (Table 2). The choice of hydrogel depends on the target application, desired properties, and operational environment. Continued innovation in hydrogel chemistry, nanomaterial integration, and device engineering will be essential to fully realize the potential of hydrogels in next-generation energy technologies.

**Table 2:** Types of Hydrogels Used in Energy Applications.

Hydrogel Type	Source/Composition	Properties	Applications	Advantages	Challenges	Ref.
Natural Hydrogels	Alginate, chitosan, cellulose	Biocompatibility, biodegradability, high gelation ability	Electrolytes, separators in batteries and supercapacitors	Renewable, environmentally friendly	Mechanical strength, scalability	[114]
Synthetic Hydrogels	Polyacrylamide (PAM), polyethylene oxide (PEO), polyvinyl alcohol (PVA)	High water content, tunable mechanical properties, high ionic conductivity	Electrolytes, separators in batteries and supercapacitors	Customizable properties, high ionic conductivity	Environmental impact, mechanical robustness	[112]
Composite Hydrogels	Graphene oxide + polymer matrix, nanoparticles + polymer matrix	Enhanced mechanical properties, high electrical conductivity	Electrodes in batteries, supercapacitors	Enhanced properties, multifunctionality	Complexity in synthesis, cost	[112]
Carbon-based Hydrogels	Graphene, carbon nanotubes (CNTs)	High electrical conductivity, mechanical strength	Electrodes in batteries, supercapacitors	High electrical conductivity, mechanical strength	Scalability, cost	[188]
Conductive Polymer Hydrogels	Polyaniline, polypyrrole	High electrical conductivity, flexibility	Electrodes, sensors, supercapacitors	High electrical conductivity, flexibility	Stability, environmental impact	[189]
Metal–Organic Framework (MOF) Hydrogels	MOFs + polymer matrix	High porosity, tunable properties	Electrolytes, gas storage, sensors	High porosity, tunable properties	Synthesis complexity, cost	[190]

## 4. Structural and Morphological Characterization

The structural and morphological properties of hydrogels are crucial in determining their performance in energy applications. Techniques such as scanning electron microscopy (SEM) and transmission electron microscopy (TEM) are widely used to analyze hydrogel porosity and microstructure [191]. Fourier-transform infrared spectroscopy (FTIR) and X-ray diffraction (XRD) help in identifying chemical compositions and crystalline structures, respectively [192]. Additionally, rheological measurements provide insights into the viscoelastic behavior of hydrogels, which is critical for their mechanical performance [193].

### 4.1. Microstructural Performance

The microstructure of hydrogels is a critical factor influencing their mechanical properties, degradation behavior, and molecular transport capabilities. Key parameters used to characterize hydrogel microstructure include the polymer volume fraction in the swollen state, mesh size, and the average molecular weight between cross-links. These structural features are influenced by the degree of crosslinking, the chemical nature of the monomers, and environmental conditions such as pH, temperature, and ionic strength [194]. Mesh size, in particular, governs the hydrogel’s mechanical strength, permeability, and ability to release encapsulated molecules. By tuning these parameters, hydrogels can be tailored for specific biomedical applications, including drug delivery, tissue scaffolding, and wound healing.

**Figure 1:**
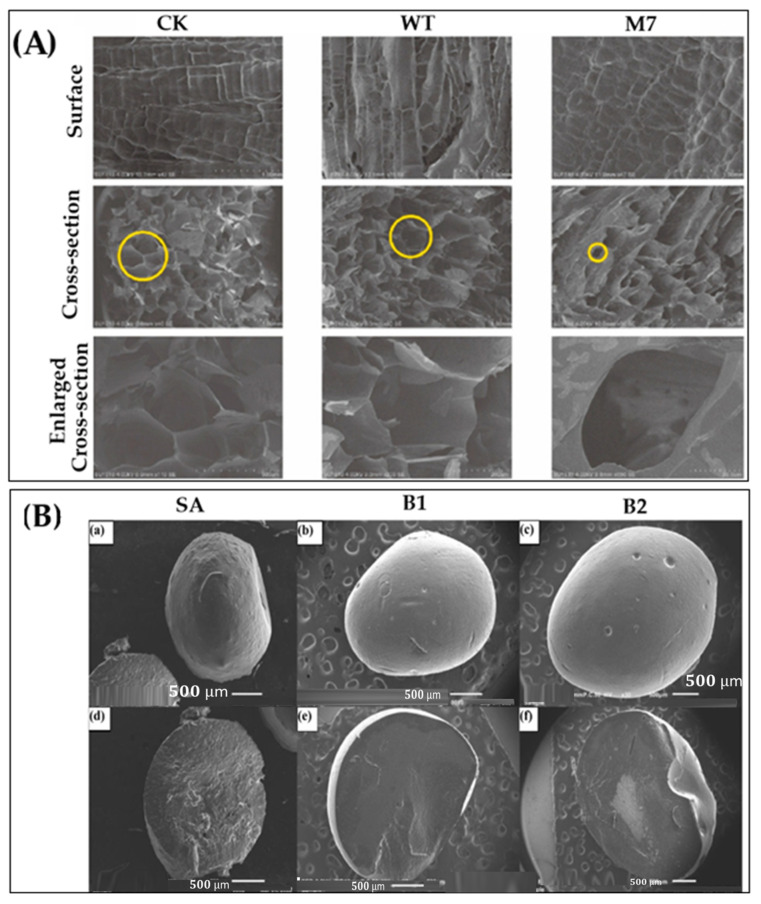
Microstructure analysis of hydrogel nanoparticles. (**A**) SEM images of natural and enzyme-induced hydrogels, where CK refers to control samples without MTG treatment, reproduced with permission from ref. [195], (**B**) external and cross-sectional views of different hydrogel beads: (**a**,**d**) SA; (**b**,**e**) and B1; (**c**,**f**) and B2. reproduced with permission from ref. [197], Elsevier.

Zhang et al. [195] used scanning electron microscopy (SEM) to examine hydrogels cross-linked with microbial transglutaminase (MTG). The M7-crosslinked variant exhibited a smoother, denser surface and a distinctive honeycomb-like internal structure, attributed to combined hydrogen bonding, hydrophobic interactions, and covalent crosslinking (Figure 1A).

Zhang et al. [195] investigated the impact of microbial transglutaminase (MTG) on hydrogel architecture, scanning electron microscopy (SEM) was employed to examine both surface and internal morphologies. The SEM images revealed notable structural differences among the samples. Specifically, the hydrogel crosslinked with M7 exhibited a significantly denser and more uniform reticulated surface compared to the native and WT-crosslinked variants, with smoother contours observed (Figure 1A). Internally, the M7-treated hydrogel displayed a well-defined honeycomb-like structure, characterized by polygonal or nearly circular pores, which were more prominent than in other samples.

These morphological enhancements are attributed to the synergistic effects of hydrogen bonding, hydrophobic interactions, and covalent crosslinking—three key forces involved in protein gelation [196]. The MTG-mediated crosslinking promotes both intermolecular and intramolecular interactions between glutamine and lysine residues, resulting in a more organized and stable network. Overall, the findings suggest that M7 crosslinking effectively improves the microstructure of hydrogels, potentially enhancing their mechanical and functional performance.

Camargo et al. [197] investigated the surface and internal morphology of sodium alginate (SA) hydrogel beads and those modified with thermosensitive materials (TSM) using scanning electron microscopy (SEM), as shown in Figure 1B. All beads exhibited an ellipsoidal shape. Compared to the SA beads (Figure 1B(a)), the SA-TSM beads (Figure 1B(b,c) had smoother surfaces and fewer visible cracks. This suggests that TSM acts as a filler material, contributing to improved surface structure and mechanical stability.

The cross-sectional SEM images (Figure 1B(d–f)) further highlight differences in internal architecture. SA beads showed a more porous and irregular structure, while SA-TSM beads (B1 and B2) demonstrated a more uniform and compact internal morphology with fewer cracks. These observations indicate that the presence of mucilage enhances structural support within the hydrogel matrix.

Similar results were reported by Lozano-Vazquez et al. [198], who studied hydrogel beads composed of SA and modified tapioca starch. Their findings confirmed that starch molecules function as filler agents, improving both surface and internal structures. Beads made solely from SA exhibited surface fissures and possible internal voids, whereas those containing starch showed a more complex and reinforced porous network. This behavior closely resembles the structural improvements observed in the SA-TSM beads analyzed in the current study.

### 4.2. Morphology Study

Morphological characterization is essential for understanding the structural and functional properties of hydrogels. Techniques such as Field Emission Scanning Electron Microscopy (FE-SEM), Environmental Scanning Electron Microscopy (ESEM), and Transmission Electron Microscopy (TEM) are commonly employed to analyze hydrogel surfaces and internal architectures.

**Figure 2:**
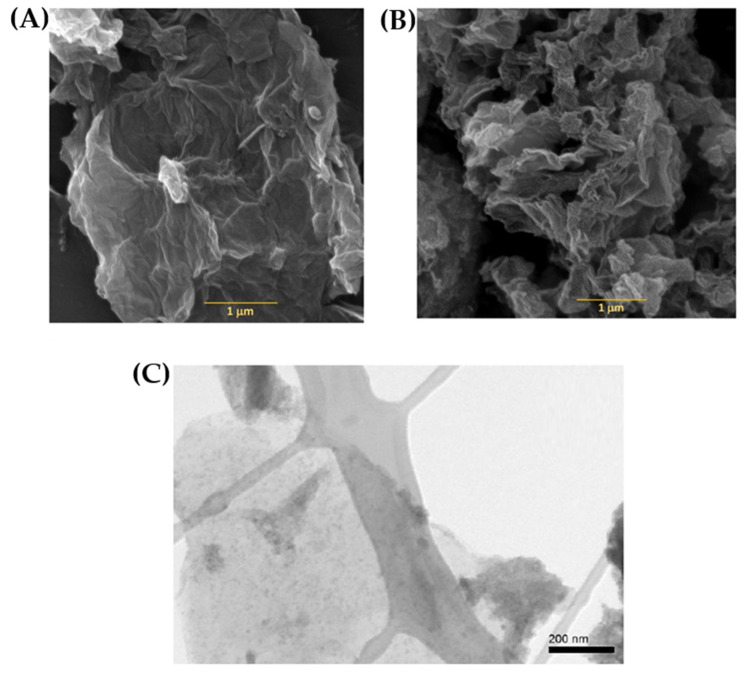
(**A**) FE-SEM image of graphene oxide (GO); (**B**) FE-SEM image of 3-(2-aminoethylamino)propyltrimethoxysilane-functionalized reduced graphene oxide (Amine-rGO); (**C**) TEM image of Amine-rGO showing thin laminated graphene layers. Adapted with permission from [200], Elsevier.

FE-SEM provides high-resolution images of dehydrated hydrogel surfaces, revealing features like porosity, roughness, and structural defects [199]. Mousavi et al. investigated the structural features of graphene-based materials and their integration into the hydrogel matrix to understand their final influence on the material properties [200]. Field emission scanning electron microscopy (FE-SEM) images of graphene oxide (GO) and 3-(2-aminoethyl amino) propyltrimethoxysilane (APTMS)-functionalized reduced graphene oxide (Amine-rGO) are shown in Figure 2A,B, respectively. GO sheets exhibited a thin, curtain-like morphology with crumpled flakes and relatively large surface areas and thicknesses. This structure reflects the deformation of nanosheets during the exfoliation and oxidation of graphite, consistent with previous findings [201].

Upon functionalization and reduction, Amine-rGO displayed fragmented and folded sheets, resulting from interactions between the functional groups of APTMS and the GO surface in the presence of an organic solvent. These sheets formed multilayered, entangled structures with ripples and irregular edges, typical of reduced graphene oxide.

Further analysis using transmission electron microscopy (TEM) (Figure 2C) revealed transparent graphene layers, confirming the formation of thin, laminated structures through the employed synthesis method.

### 4.3. Mechanical Properties and Performance of Hydrogels

The mechanical behavior of hydrogels is a critical factor in determining their suitability for biomedical and engineering applications. These properties are typically evaluated using standard techniques such as tensile, compression, indentation, bulge, and cyclic testing. Tensile tests provide insights into elasticity and strength, while compression tests assess deformation under load. Indentation and bulge tests help characterize localized stiffness and membrane behavior, respectively. Cyclic or fatigue testing evaluates durability under repeated stress, which is essential for long-term applications.

**Figure 3:**
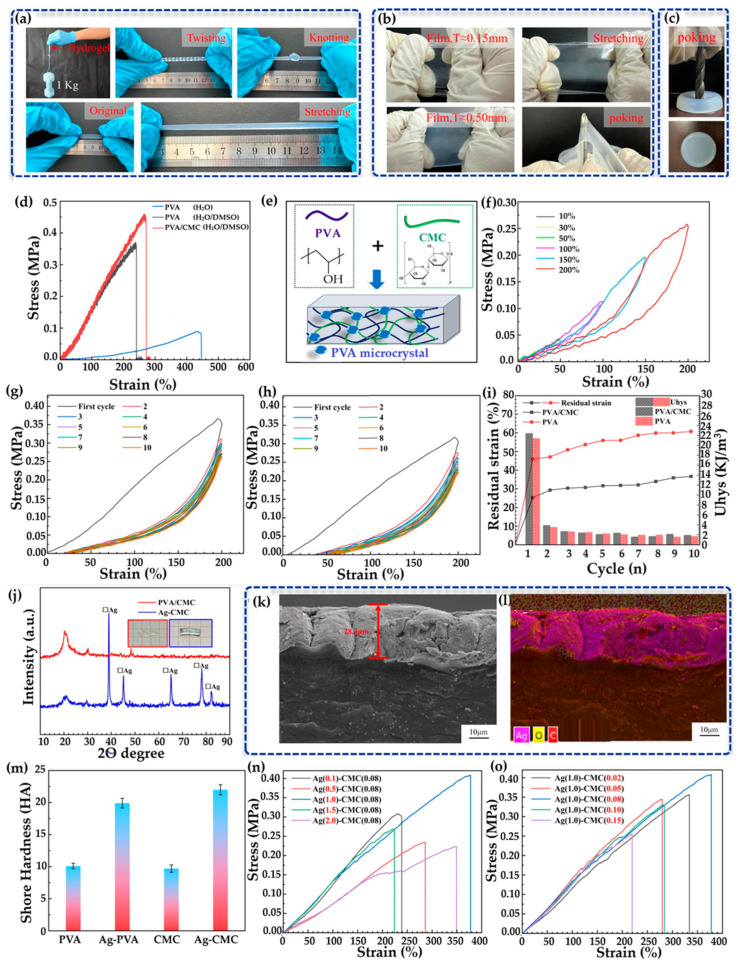
(**a**) Mechanical flexibility of PVA/CMC hydrogel demonstrated through hanging, twisting, knotting, and stretching. (**b**,**c**) Stretching and puncture resistance of PVA/CMC hydrogel film and block, respectively. (**d**) Tensile stress–strain profiles of PVA-CMC (H_2_O/DMSO), pure PVA (H_2_O/DMSO), and pure PVA (H_2_O) hydrogels. (**e**) Schematic of freeze-molded CMC and PVA chain arrangement. (**f**) Loading–unloading behavior of PVA-CMC hydrogels under varying strain levels. (**g**–**i**) Cyclic loading–unloading, residual strain, and energy dissipation of PVA-CMC and pure PVA hydrogels. (**j**) XRD patterns of PVA/CMC and Ag-CMC conductive hydrogels. (**k**,**l**) Cross-sectional morphology of Ag-CMC conductive hydrogels. (**m**) Shore hardness of PVA and PVA-CMC hydrogels before and after silver reduction. (**n**,**o**) Tensile stress–strain curves of conductive hydrogels with varying AgNO_3_ and VC concentrations, reproduced with permission from ref. [202]), Elsevier.

Hou et al. [202] demonstrated that PVA-CMC hydrogels possess excellent mechanical performance for soft strain sensors. Cyclic stress–strain tests showed consistent behavior across multiple loading cycles, confirming their durability and elasticity (Figure 3a–o). PVA-based hydrogels demonstrate remarkable mechanical resilience, especially when modified with additives like DMSO and carboxymethyl cellulose (CMC). A hydrogel strip (3.5 mm thick, 4.5 mm wide) was able to support 1 kg without damage and maintained its integrity under stretching, twisting, knotting, and drilling (Figure 3a–d).

The addition of DMSO reduced the fracture strain of pure PVA from 446.48% to 241.18%, while increasing its fracture stress to 0.366 MPa. Incorporating CMC further enhanced both strength and strain, reaching 0.453 MPa and 272.13%, respectively (Figure 3e). Cyclic testing revealed stable hysteresis behavior, indicating excellent fatigue resistance and energy dissipation, particularly due to increased crosslinking density from DMSO (Figure 3f–i). Hardness tests showed that Ag-CMC hydrogels had superior values compared to pure PVA, and the Ag(1.0)-CMC(0.08) formulation achieved optimal mechanical strength and low electrical resistance (Figure 3f,m–o).

### 4.4. Fourier Transform Infrared Spectroscopy (FTIR)

FTIR spectroscopy is a powerful analytical technique used to identify the chemical structure and bonding characteristics of hydrogel materials. It works by detecting the absorption of infrared light at specific frequencies corresponding to the vibrational modes of chemical bonds. Each functional group within a molecule absorbs IR radiation at characteristic frequencies, allowing researchers to determine the presence and interactions of these groups within hydrogel systems [203].

**Figure 4:**
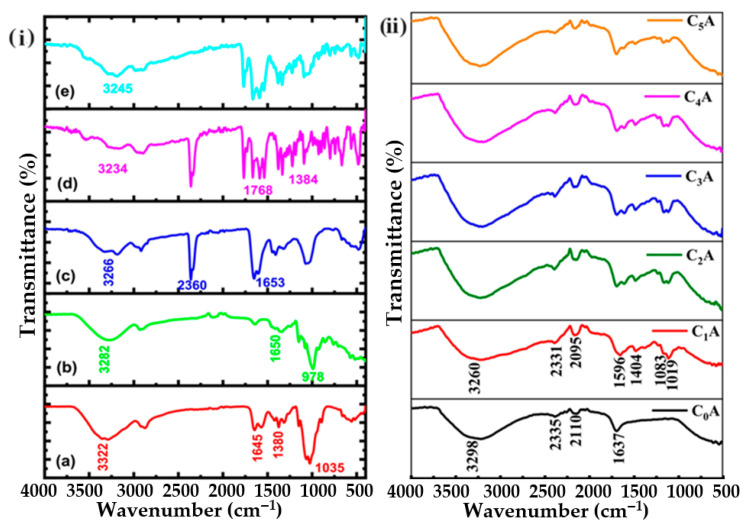
(**i**) FTIR spectra illustrating the characteristic peaks of (**a**) pure chitosan, (**b**) native starch, (**c**) chitosan–starch composite hydrogel, (**d**) cefixime, and (**e**) cefixime-incorporated hydrogel, reproduced with permission from ref. [204], Elsevier. (**ii**) FTIR profiles of chitosan/alginate hydrogel systems, reproduced with permission from ref. [205], Elsevier.

Medha et al. [204] analyzed FTIR spectra of chitosan, starch, chitosan-starch hydrogel, and cefixime-loaded hydrogel. Broad absorption bands between 3300 and 2850 cm^−1^ were observed, corresponding to –OH, –NH_2_, and –CH stretching vibrations. Chitosan exhibited peaks at 1645 cm^−1^ (C=O stretching), 1548 cm^−1^ (–NH bending), and 1380 cm^−1^ (–CH_3_ deformation), while starch showed weaker signals at 1650 cm^−1^ and deformation bands at 1436 and 1340 cm^−1^. The chitosan-starch hydrogel displayed a band at 1653 cm^−1^, indicating successful grafting and crosslinking. Cefixime-loaded hydrogels showed distinct peaks at 1768, 1669, 1588, 1537, and 1384 cm^−1^, suggesting drug-polymer interactions (Figure 4i(a–e)).

Enoch et al. [205] further investigated chitosan/alginate hydrogels at varying concentrations. The alginate hydrogel (C0A) showed peaks at 3298 cm^−1^ (O–H stretching), 2110 cm^−1^ (C–H stretching), 2335 cm^−1^ (CO_2_), and 1637 cm^−1^ (COO^−^ stretching). Chitosan/alginate composites (C1A to C5A) exhibited overlapping O–H and N–H stretching bands around 3260 cm^−1^, with additional peaks at 1019 and 1083 cm^−1^ (C–O and C–H stretching), and 1404 and 1596 cm^−1^ indicating amine-aldehyde interactions and COO^−^ stretching (Figure 4ii).

### 4.5. Viscoelastic Properties

Viscoelasticity is a defining mechanical characteristic of hydrogels, reflecting their ability to exhibit both elastic and viscous responses under deformation. These properties are typically assessed through rheological methods such as compression testing and oscillatory shear analysis. The viscoelastic behavior of hydrogels can be finely tuned by adjusting polymer and crosslinker concentrations, which directly influence stiffness, elasticity, and energy dissipation [206].

**Figure 5:**
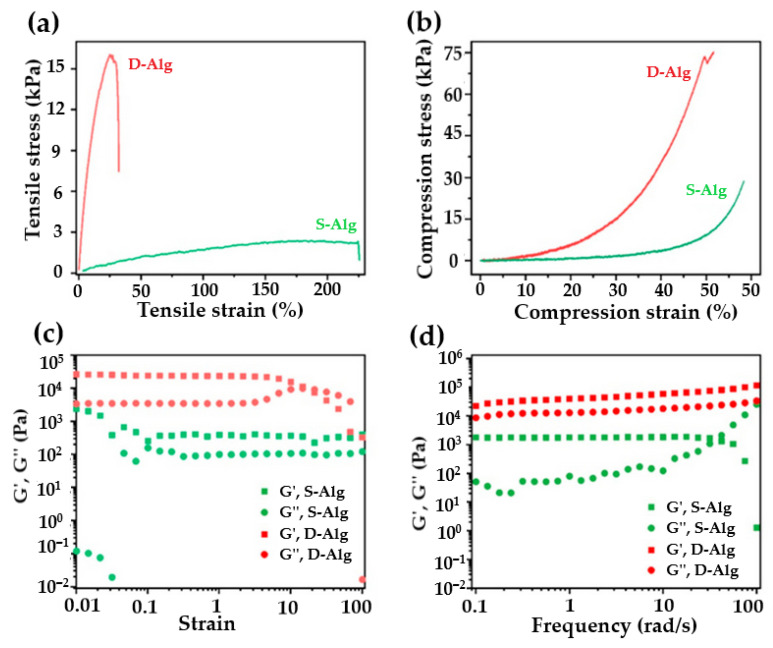
Mechanical and rheological characterization of S–Alg and D–Alg hydrogels (**a**) tensile, (**b**) compression, (**c**) amplitude, and (**d**) frequency sweep tests, reproduced with permission from ref. [207], Elsevier.

Zhang et al. [207] demonstrated that S-Alg hydrogels exhibited low tensile modulus (2.7 ± 0.7 kPa) but high elongation (224%), whereas D-Alg hydrogels showed significantly higher tensile (134.6 ± 6.2 kPa) and compression modulus (453.9 ± 16.9 kPa), indicating enhanced stiffness. Viscoelastic analysis revealed that both hydrogels maintained a wide linear viscoelastic range up to 10% strain, with D-Alg displaying a higher storage modulus (G′) than loss modulus (G″), consistent with robust network structures (Figure 5a–d).

For example, increasing the concentration of glutaraldehyde in gelatin hydrogels enhances stiffness and shifts the material toward a more elastic regime. Similarly, modifying the viscosity of the aqueous phase with dextran in agarose or polyacrylamide hydrogels allows for control over viscoelasticity while maintaining a stable elastic modulus—an important consideration for biomedical applications.

In physically or non-covalently crosslinked hydrogels, viscoelasticity arises from dynamic molecular mechanisms. Crosslinkers may temporarily detach under stress, allowing the polymer matrix to flow and then reattach, as observed in collagen, alginate, and PEG-based gels. Additional contributions from polymer entanglement and protein unfolding further enhance energy dissipation and reversible deformation [208]. Even in well-crosslinked systems, the high water content contributes to a measurable loss modulus, enabling viscoelastic behavior without permanent deformation.

## 5. Hydrogel-Based Materials in Battery Applications

Hydrogels have garnered significant attention in recent years as multifunctional materials for advanced battery technologies. Their soft, water-rich polymer networks offer unique advantages such as high ionic conductivity, mechanical flexibility, and environmental compatibility, making them ideal candidates for next-generation energy storage systems. This section delves into the synthesis and integration of hydrogel-based components within battery architectures, highlighting their role in improving performance, safety, and sustainability.

### 5.1. Synthesis and Fabrication of Hydrogel Battery Components

The integration of hydrogels into battery systems requires a tailored synthesis approach that ensures compatibility between the hydrogel matrix and electrochemical components [209]. Unlike traditional battery fabrication, hydrogel-based systems demand careful control over both the chemical composition and physical architecture to optimize performance [210].

#### 5.1.1. Fabrication of Hydrogel Electrolytes

Hydrogel electrolytes are typically synthesized through in situ polymerization of hydrophilic monomers such as acrylamide, polyvinyl alcohol (PVA), or polyethylene glycol (PEG), often in the presence of crosslinkers (e.g., N,N′-methylenebisacrylamide) and initiators (e.g., ammonium persulfate) [211,212]. After gelation, the hydrogel is immersed in an electrolyte solution (e.g., LiPF_6_, ZnSO_4_, or AlCl_3_) to enable ionic conductivity [34]. The resulting gel exhibits high water content, mechanical flexibility, and tunable ionic transport properties.

#### 5.1.2. Integration with Electrodes

To enhance electrochemical performance, hydrogel matrices can be embedded with conductive fillers such as graphene oxide (GO), reduced graphene oxide (rGO), carbon nanotubes (CNTs), or metal oxides. These composites are either formed during the polymerization process or by post-synthesis blending. For example, Amine-functionalized rGO incorporated into the hydrogel matrix improves both electrical conductivity and mechanical integrity [200].

#### 5.1.3. Assembly of Full Cells

The hydrogel-based electrolyte or electrode is cast onto current collectors (e.g., stainless steel, carbon cloth, or copper foil) and dried under controlled conditions [209]. In some designs, the hydrogel serves as both the electrolyte and separator, simplifying the architecture and improving flexibility [93,213]. The cathode and anode are then assembled with the hydrogel layer in between, and the full cell is sealed for electrochemical testing.

### 5.2. Functional Properties and Mechanisms

#### 5.2.1. Hydrolytic Behavior and Water Absorption

Hydrogels exhibit exceptional water absorption due to the presence of hydrophilic functional groups—such as hydroxyl (–H), carboxyl (–COOH), carbonyl (–C=O), amine (–NH_2_), amide (–CONH_2_), and sulfonic acid (–SO_3_H)—along their polymer backbone [214]. Upon exposure to water, these polar groups facilitate the uptake of water molecules, forming primary bound water (PBW). This initial swelling expose hydrophobic domains, which subsequently interact with water to form secondary bound water (SBW). The total bound water comprises both PBW and SBW. Further absorption is driven by osmotic pressure gradients within the polymer network, leading to the uptake of free or unbound water (UB), which occupies the interstitial spaces between polymer chains. The swelling process is ultimately constrained by the network’s crosslinking density, which generates an elastic retraction force opposing further expansion [215]. Water retention within hydrogels significantly influences their mechanical integrity, solute diffusion, and electrochemical properties. Factors such as the concentration of hydrophilic groups, crosslinking density, and environmental conditions (e.g., pH, temperature, ionic strength) modulate this [216,217]. These characteristics make hydrogels particularly suitable for applications in wearable energy devices, including batteries, where their high water content supports ion mobility and conductivity [218].

#### 5.2.2. Porosity and Ion Transport

Hydrogels possess a porous three-dimensional network that facilitates efficient ion migration, making them viable alternatives to eutectic electrolytes in energy storage systems [219,220]. Their nanostructured porosity enhances electrochemical performance by increasing the active surface area, improving solid–liquid interfaces, and shortening ion transport pathways [219]. This structural advantage also enables hydrogels to accommodate mechanical strain during repeated charge–discharge cycles, thereby enhancing device durability. Moreover, the water-fixing ability of hydrogels suppresses side reactions such as hydrogen evolution and dendrite formation, particularly in high-voltage aqueous batteries [221].

#### 5.2.3. Safety and Non-Toxicity

Hydrogels offer superior safety profiles compared to organic solvent-based electrolytes due to their aqueous nature. Their quasi-solid structure mitigates risks associated with liquid leakage and water-splitting reactions, while also suppressing dendrite growth and electrode dissolution [32,33,221]. These properties make hydrogels particularly attractive for applications requiring high reliability and environmental compatibility, such as wearable electronics and biomedical devices.

#### 5.2.4. Self-Healing Capability

Certain hydrogel systems exhibit intrinsic self-healing behavior, which is crucial for extending the operational lifespan of flexible energy storage devices [222,223]. This self-repair mechanism is typically governed by dynamic covalent or reversible non-covalent interactions—such as hydrogen bonding, ionic bonding, or coordination chemistry—that allow the polymer network to autonomously reconfigure after mechanical damage [224]. For instance, self-healing hydrogel electrolytes have demonstrated the ability to restore electrochemical performance after being physically severed, with recovery efficiencies exceeding 90% [225]. This property distinguishes hydrogels from conventional separators (e.g., ceramic or polymer membranes), which lack such regenerative capabilities.

#### 5.2.5. Tunable Mechanical Properties and Design Flexibility

Hydrogels can be engineered to exhibit a wide range of mechanical properties, including tunable Young’s modulus from kilopascals to megapascals, depending on factors such as monomer concentration, crosslinking chemistry, and entanglement density [30,226]. The mechanical behavior of highly entangled hydrogels, for example, can be modulated by controlling the distribution and mobility of polymer entanglements, which significantly affects elasticity and toughness [227]. Additionally, incorporating functional additives—such as conductive polymers, salts, or plasticizers—can impart multifunctionality, including enhanced electrical conductivity and thermal stability [228,229].

Hydrogels also offer other advantageous properties for battery applications, including biocompatibility, permeability, dimensional stability, and environmental sustainability. Their biodegradability and low-cost synthesis further support the development of eco-friendly energy technologies.

### 5.3. Advantages and Challenges

Hydrogel-based battery systems offer several advantages:

- Mechanical flexibility for wearable and stretchable electronics [120,230].
- Improved safety due to non-flammable, water-based electrolytes [230].
- Enhanced interface stability and ion transport [231,232].

However, challenges remain in optimizing long-term stability, preventing water evaporation, and ensuring compatibility with high-voltage electrodes [233].

In the following sections, we explore how hydrogel-based materials are being applied across battery components, highlighting their role in hydrogel-based electrodes, binders, electrolytes, and other hydrogel-derived multifunctional materials.

## 6. Hydrogels in Specific Battery Chemistries

Rechargeable batteries are central to modern energy storage, typically composed of a cathode, anode, and electrolyte. These components work together to store and transfer charge carriers—commonly alkaline metal ions like Li^+^, Na⁺, Zn^2+^, Mg^2+^ and Al^3+^—during charge and discharge cycles. These ions are favored for their high reactivity, low reduction potential, and high specific capacity, enabling efficient energy conversion and storage.

However, conventional lithium-ion batteries are nearing their performance limits, with energy densities below 500 Wh kg^−1^, rising material costs, and persistent safety concerns [234]. To address these issues, researchers have explored multivalent ions such as Mg^2^⁺, Ca^2^⁺, Zn^2^⁺, and Al^3+^, which offer higher charge densities and lower cost. Yet, practical implementation remains limited due to challenges in ion mobility and electrode compatibility [32,235].

Hydrogels have emerged as a promising solution to these limitations. Their unique multifunctionality—including easy fabrication [236], tunable ionic and electronic conductivity [236,237], ability to form stable solid–electrolyte interphases [238,239], and enhanced mechanical strength [240,241]—makes them ideal candidates for improving battery performance.

Hydrogels have emerged as promising materials to overcome these limitations due to their multifunctional nature. Their selection for battery applications is guided by three key design principles:

- Molecular Backbone: Synthetic polymers such as polyvinyl alcohol (PVA), polyethylene oxide (PEO), and acrylamide are commonly used due to their hydrophilicity and ability to coordinate with metal ions, facilitating ion transport. Natural polymers like alginate, chitosan, and gelatin offer biocompatibility and biodegradability, making them ideal for wearable and implantable devices [33,127,147,242,243,244,245].
- Functional Groups: The presence of hydroxyl (–OH), carboxyl (–COOH), amine (–NH_2_), and sulfonic acid (–SO_3_H) groups enables chemical modification, ion coordination, and strong interfacial adhesion with electrodes and current collectors [246,247].
- Extent of Crosslinking: Hydrogels can be physically crosslinked (via hydrogen bonding or ionic interactions), chemically crosslinked (via covalent bonding using agents like glutaraldehyde or borax), or radiation crosslinked (using gamma or electron beams). The degree and type of crosslinking determine the hydrogel’s mechanical strength, swelling behavior, and electrochemical stability [248,249].

**Figure 6:**
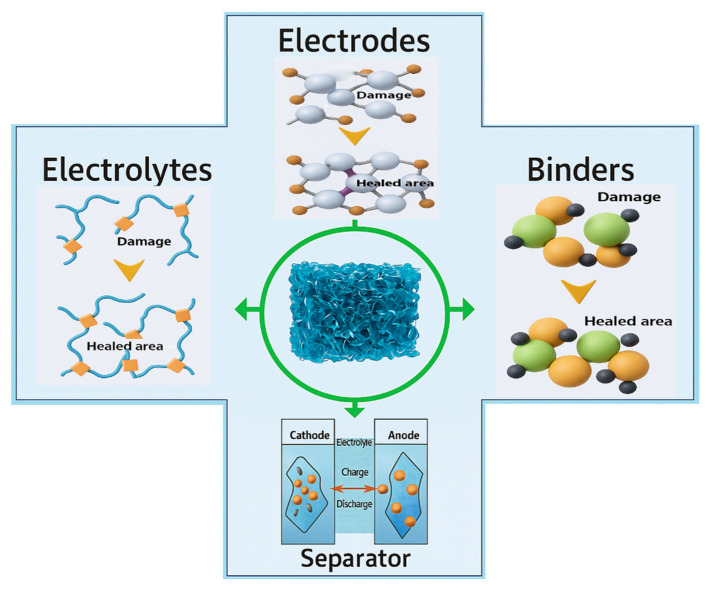
3D conceptual diagram showing hydrogel-based materials functioning as electrodes, binders, electrolytes, and separators in advanced battery systems, adapted and redrawn from sources [260], Springer Nature 2024.

These properties allow hydrogels to function as electrodes [250], binders [250,251,252,253], electrolytes [254,255], and separators [256,257,258,259], offering improved charge transport, mechanical integrity, and interface stability (Figure 6) [260]. Their ability to integrate with conductive fillers and nanomaterials further enhances their performance in next-generation battery systems.

### 6.1. Hydrogels in Li-Ion Batteries

#### 6.1.1. Hydrogel-Derived Electrodes

Electrodes are the core components of rechargeable batteries, where redox reactions facilitate energy storage and release. For optimal performance, electrodes must support rapid ion and electron transport, maintain mechanical integrity, and ensure strong connectivity between active materials and current collectors. However, conventional electrode fabrication methods—such as solid-state reactions and high-temperature annealing—often result in particle agglomeration, poor dispersion, and irregular morphologies, which hinder conductivity and ion diffusion [261].

**Figure 7:**
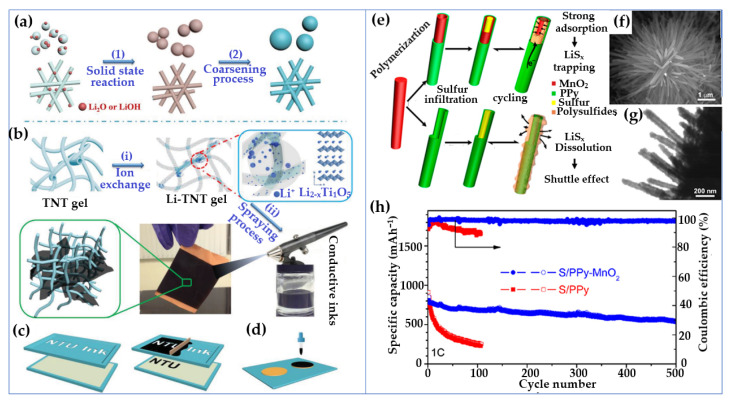
(**a**) Schematic illustration of conventional solid-state synthesis of LTO (L_i4_Ti_5_O_12_) involves mixing lithium oxide with TiO_2_-based materials followed by high-temperature annealing; (**b**) In situ synthesis of conductive lithium titanate nanotube (Li-TNT ink): (**i**) ion-exchange to form Li-TNT precursor; (**ii**) spray-coating onto Cu foil, converting to LTO/RGO after thermal treatment; (**c**,**d**) Screen-printing and drop-casting processes for applying conductive Li-TNT inks; adapted with permission from ref. [262], Wiley & Sons. (**e**) Schematic of S/PPy–MnO_2_ composite synthesis and its electrochemical benefits during cycling. (**f**,**g**) SEM and TEM images showing MnO_2_ nanowires and PPy–MnO_2_ nanotubes, respectively. (**h**) Comparison of sulfur electrodes encapsulated with PPy–MnO_2_ nanotubes versus pure PPy nanotubes, adapted with permission from ref. [263], copyright© 2016 American Chemical Society.

Hydrogels offer an innovative solution to these limitations by serving as multifunctional platforms for electrode design. Their three-dimensional polymeric networks provide a uniform and porous matrix that enables homogeneous dispersion of active materials and conductive additives. In the work by Tang et al. [262], a hydrogel-based ink composed of Li_4_Ti_5_O_12_ (LTO) and reduced graphene oxide (rGO) was developed, allowing for solution-processable coating onto current collectors (Figure 7a–d). The hydrogel facilitated the formation of a continuous and conductive framework, where rGO nanosheets bridged LTO particles and the substrate, enhancing electron transport and mechanical cohesion.

Moreover, the hydrogel’s mesoporous structure shortened Li-ion diffusion paths and buffered volume changes during cycling, improving rate capability and structural stability. Unlike traditional binders, hydrogels can be engineered to possess ionic conductivity, self-healing properties, and mechanical flexibility, making them ideal for next-generation battery architectures, including flexible and wearable devices.

Hasegawa et al. [264] made a significant contribution by synthesizing meso-/macroporous LiFePO_4_ through a polymer-assisted method using metal precursors and polymers such as PEO and polyvinylpyrrolidone. The resulting porous architecture provided a high surface area, enhancing reaction kinetics and charge transfer—particularly beneficial for low-conductivity materials like LiFePO_4_. Additionally, the carbon coating derived from polyvinylpyrrolidone improved electronic conductivity, leading to superior electrochemical performance. Similar synthesis strategies have also been applied to other nanoscale electrode materials, including MnO_2_, TiO_2_, and Fe_2_O_3_ [265,266,267,268].

Beyond geometric advantages, hydrogel-based composites offer solutions for more complex systems like lithium–sulfur (Li–S) batteries, which promise high specific capacities (sulfur: 1675 mAh g^−1^; Li metal: 3860 mAh g^−1^). However, sulfur’s poor conductivity, polysulfide dissolution, and volume-change degradation remain major challenges [269,270]. To mitigate these issues, Zhang et al. [263] designed a composite hydrogel-derived electrode using MnO_2_ nanowires as oxidants for polypyrrole (PPy) polymerization (Figure 7e–h). After sulfur infiltration, the resulting S/PPy–MnO_2_ electrode exhibited excellent Coulombic efficiency, cycling stability, and rate capability (Figure 7d). The MnO_2_ nanowires effectively trapped polysulfides, while the conductive PPy hydrogel facilitated rapid electron transport.

**Figure 8:**
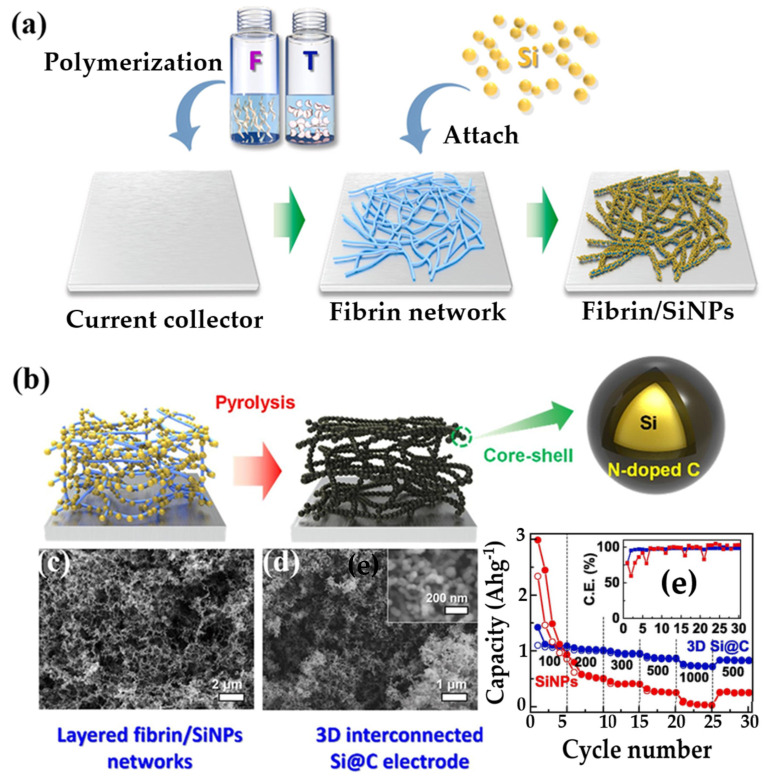
(**a**) Schematic of the fibrin/SiNPs assembly, (**b**) 3D Si@C electrodes, (**c**,**d**) SEM/TEM images before and after pyrolysis, respectively, (**e**) Rate performances of 3D Si@C electrode and conventional SiNPs mixture electrode at 100–1000 mA g^−1^. Closed and open symbols denote lithiation and delithiation specific capacities, respectively. Inset shows the corresponding Coulombic efficiency (C.E.), reproduced with permission from ref. [271], Elsevier.

Hydrogels have also shown promise in addressing the challenges associated with silicon anodes, which offer high theoretical capacity but suffer from severe volume expansion and poor cycling stability. A novel strategy proposed by [271] utilized a fibrin biopolymer hydrogel as a template to fabricate a three-dimensional (3D) interconnected Si@C framework (Figure 8a).

This binder- and additive-free architecture utilizes strong hydrogen bonding between fibrin amide groups and hydroxyl-functionalized silicon nanoparticles (SiNPs), enabling uniform dispersion of SiNPs within a nitrogen-doped amorphous carbon matrix. Pyrolysis of the hydrogel preserves the porous 3D structure and introduces pyridinic and pyrrolic nitrogen species, which enhance both electronic conductivity and lithium storage capability (Figure 8 b–d). The resulting 3D Si@C electrode exhibited discharge capacities of 1090, 1015, 955, and 870 mAh g^−1^ at current densities of 100 (0.05C), 200 (0.1C), 300 (0.15C), and 500 (0.25C) mA g^−1^, respectively. Notably, it retained a capacity of 725 mAh g^−1^ even at a higher current density of 1000 mA g^−1^ (0.5C). Upon returning to 500 mA g^−1^, the capacity recovered to 836 mAh g^−1^, corresponding to 96% of its initial value at that rate, indicating excellent rate capability and reversibility (Figure 8e). This scalable and sustainable design offers a promising route for advanced electrode architectures, with further optimization possible through control of fibrin mesh size, Si loading, and carbon crystallinity. In addition to its role as a structural template, this system also functions as a binder-free architecture due to the intrinsic bonding interactions.

#### 6.1.2. Hydrogel-Derived Binders

**Figure 9:**
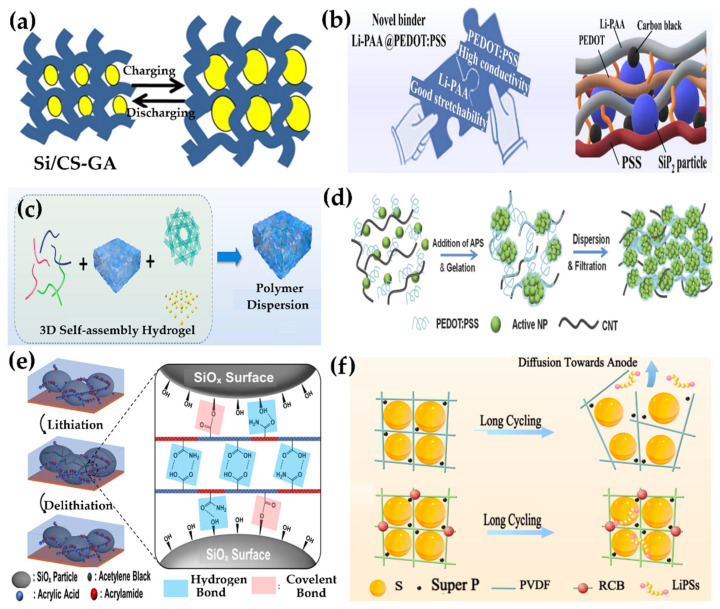
Schematic representations of various hydrogel-based binder systems designed for silicon anodes, highlighting in situ polymerization approaches and structural strategies to mitigate volume expansion during cycling. (**a**) Charge/discharge mechanism of Si/CS-GA anodes, adapted with permission from ref. [276], copyright © 2016, American Chemical Society; (**b**–**f**) diverse hydrogel architectures including CHSP and RCB binders, demonstrating enhanced mechanical integrity and electrochemical performance. Adapted with permission from ref. [277,278] Elsevier; ref. [229], Wiley & Sons; [279,280] American Chemical Society.

The rising demand for high-energy-density batteries in electric vehicles, wearable electronics, and grid storage has intensified the need for electrodes with high capacity, flexibility, and long-term cycling stability [272,273,274]. However, silicon (Si) and sulfur-based electrodes suffer from severe volume expansion during lithiation/delithiation, leading to mechanical degradation, poor conductivity, and rapid capacity fading [275]. To address these challenges, various research groups have developed hydrogel-based binders and carbon frameworks that offer mechanical flexibility, strong adhesion, and the ability to accommodate volume changes. One notable example is the hydrogel binder development. A chitosan (CS)-based binder crosslinked with glutaraldehyde (GA), shown in Figure 9a, forms a robust 3D network that enhances mechanical stability. This system delivered 2782 mAh g^−1^ initially and maintained 1969 mAh g^−1^ after 100 cycles [276]. It is worth noting that the 3D Si@C framework discussed in Section 6.1.1 also inherently serves as a binder-free system, leveraging strong hydrogen bonding between fibrin and silicon nanoparticles to maintain structural integrity without the need for additional binder materials.

In another approach, Liu et al. [277] developed a hierarchical hydrogel framework (Figure 9b) by incorporating a conducting polymer. This 3D network provided continuous electrical pathways and space for Si expansion, achieving over 90% capacity retention after 5000 cycles at 6.0 A g^−1^. To further enhance flexibility and conductivity

A double-network hydrogel composed of polyvinyl alcohol (PVA) and sodium alginate (SA), reinforced with hydroxypropyl cellulose (HPC), formed a porous structure (Figure 9c) that buffered Si expansion and improved ion transport [278].

Beyond these, Chen et al. [229] introduced a hybrid hydrogel system combining carbon nanotubes (CNTs) and a conductive polymer (Figure 9d). This design improved mechanical strength and ion transport, demonstrating excellent performance in both TiO_2_ and SiNP electrodes. A copolymer binder made from acrylamide and acrylic acid (Figure 9e) formed a covalent–noncovalent elastic network, significantly enhancing the capacity of SiOx anodes to 734 mAh g^−1^ after 300 cycles. Polyimine binders also showed promise, achieving 804.4 mAh g^−1^ with 82.4% retention after 1000 cycles, and 2114 mAh g^−1^ over 200 cycles in high-loading Si electrodes [279].

Mu et al. [280] developed a responsive/confinement network blend (RCB) binder (Figure 9f) using hyaluronic acid and a tetrazole-functionalized copolymer. This dual-network structure mimics muscle tissue, coordinating mechanical stress and accommodating sulfur expansion. Even with just 5 wt% binder, the system showed excellent cycling and rate performance under high sulfur loading. Inspired by spider webs, a tapioca–PAA (TA–PAA) binder was developed with high viscoelasticity and self-healing properties. This binder enabled a SiO anode to deliver 901.2 mAh g^−1^ over 1200 cycles and 619.2 mAh g^−1^ at a high rate of 5 A g^−1^ [281]. Additionally, an alginate hydrogel crosslinked with Ca^2^⁺ ions improved the performance of Si/C anodes. For wearable electronics, Wang et al. [282] created an ultrasoft, all-hydrogel coaxial fiber battery with a low Young’s modulus, achieving 84.8 mAh g^−1^ at 0.5 A g^−1^ and maintaining stable performance under deformation.

In parallel, lithium–sulfur (Li–S) batteries face their own set of challenges, including sulfur’s poor conductivity and up to 80% volume expansion during cycling [283]. 

Hydrogels have also been explored as carbon sources. Liu et al. [284] fabricated a carbon–MOF composite electrode with ZIF-67 and HKUST-1, achieving an areal capacity of over 16 mAh cm^−^^2^ and 82% retention after 300 cycles. Xu et al. [285] embedded Sn nanoparticles in hydrogel-derived carbon (Sn@PHDC), which improved lithium-ion diffusion, reduced charge transfer resistance, and enhanced cycling stability.

Together, these innovations highlight the versatility and effectiveness of hydrogel-based binders and frameworks in addressing the mechanical and electrochemical limitations of both Si and sulfur electrodes. They offer scalable, multifunctional solutions for the development of next-generation, high-performance energy storage systems.

#### 6.1.3. Hydrogel Electrolytes for Aqueous Lithium-Ion Batteries

Hydrogels have attracted considerable interest in lithium-ion battery (LIB) research due to their tunable porous structures, which facilitate lithium-ion diffusion and reduce transport resistance [32,286,287]. These properties enable rapid ion transport and stable electrode–electrolyte interfaces, enhancing overall electrochemical performance. Hydrogels can also be engineered to exclude free water, improving compatibility with electrodes and current collectors, and are increasingly used as solid electrolytes and separators in aqueous LIBs [288,289]. Aqueous systems offer improved safety and environmental compatibility, but are limited by the narrow electrochemical stability window of water (~1.23 V), which constrains energy density [290].

**Figure 10:**
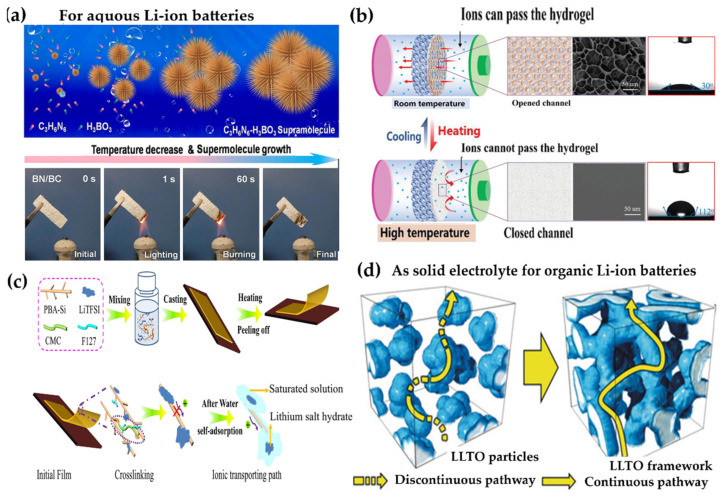
Schematics illustrating various hydrogel-based electrolyte systems developed for energy storage applications, emphasizing their structural configurations and functional contributions to ionic transport and mechanical stability. Panels (**a**–**d**) depict representative designs reported in the recent literature. Adapted with permission from ref. (**a**) [259], Elsevier; (**b**) [258], Wiley & Sons.; (**c**) [230], Elsevier; and (**d**) [292], Wiley & Sons.

To address these limitations, researchers have developed functional hydrogels tailored for specific challenges such as flame retardancy, thermal protection, mechanical flexibility, and electrochemical stability. Yang et al. [259] created a flame-retardant separator using a boron nitride (BN) aerogel synthesized from a melamine–boric acid (MBA) supramolecular hydrogel (Figure 10a). Combined with bacterial cellulose (BC), the BN/BC composite exhibited excellent flame resistance, electrolyte wettability, and mechanical strength. When used in LIBs, it delivered a high specific discharge capacity of 146.5 mAh g^−1^ and showed only 0.012% capacity degradation per cycle over 500 cycles.

To improve high-temperature safety, thermos-responsive hydrogel separators were developed by polymerizing hydrogel layers on hydrophilic membranes. These smart separators block lithium-ion transport at elevated temperatures and reopen upon cooling. By adjusting lithium salt concentration (e.g., 1 M LiNO_3_), the shut-off temperature was tunable between 30 and 80 °C (Figure 10b). A self-protecting LiMn_2_O_4_/carbon-coated LiTi_2_(PO_4_)_3_ battery using this separator demonstrated stable operation in high-temperature environments [258].

Stretchable battery components have also been realized using crumpled-structured nanowires (NWs) and crosslinked hydrogels. These materials form interconnected wrinkled NWs and robust hydrogel interfaces that maintain electrochemical stability under strain. A fully stretchable LIB (FSSLIB) using this design achieved 119 mAh g^−1^ capacity and retained 91.6% of its capacity after 250 stretching cycles at 100% strain [291].

Incorporating self-healing into stretchable systems, freestanding hydrogel electrolyte films (HEFs) were fabricated using hygroscopic lithium salts and dynamic polymer networks (PBA-Si, CMC, F127, and LiTFSI) (Figure 10c). These HEFs absorbed ambient moisture to form ionic pathways, exhibited 1.15 mS cm^−1^ conductivity, and stretched up to 300% of their original length. A full cell using HEF with NTCDA and LiMn_2_O_4_ retained 73.2% capacity after 1000 cycles at 1 A g^−1^, demonstrating excellent mechanical and electrochemical performance for wearable applications [230].

Beyond soft materials, hydrogels have been used as templates for solid-state frameworks. A 3D nanostructured Li_0.35_La_0.55_TiO_3_ (LLTO) framework derived from hydrogel precursors significantly improved Li-ion conductivity (8.8 × 10^−^^5^ S cm^−1^ at room temperature), serving as a nanofiller in composite polymer electrolytes. Similarly, Bae et al. [292] used nanostructured hydrogels to fabricate 3D interconnected garnet frameworks of Li_6_._28_La_3_Zr_2_Al_0.24_O_12_ (LLZO), achieving high conductivity (~10^−^^3^ S cm^−1^ at 60 °C) and excellent interfacial stability with lithium metal (Figure 10d).

A critical challenge in aqueous LIBs is the direct contact between water-containing electrolytes and lithium metal anodes, which can lead to violent reactions or combustion [293,294]. To address this, a dense lithium-conducting interlayer, such as a ceramic Li-ion conductor (e.g., Li_7_La_3_Zr_2_O_12_ or Li_1_._5_Al_0.5_Ge_1_._5_(PO_4_)_3_), is often introduced between the hydrogel electrolyte and the lithium anode [295]. This interlayer spatially separates the water-based hydrogel from the reactive lithium surface, preventing adverse reactions while allowing efficient lithium-ion transport. In designs where such a ceramic layer is not used, the hydrogel interface must be carefully engineered to suppress free water content and stabilize the water–Li interface. Strategies include using water-in-salt electrolytes, incorporating hydrophobic polymer chains, or forming protective solid electrolyte interphases (SEIs) that shield the lithium surface [296,297,298]. These advancements highlight the versatility of hydrogel-based materials in addressing the mechanical, thermal, and electrochemical challenges of next-generation lithium-ion batteries, particularly for flexible, wearable, and high-safety applications.

#### 6.1.4. Hydrogels Under Extreme Conditions

Hydrogels are increasingly used in battery systems due to their excellent ionic conductivity, mechanical flexibility, and compatibility with aqueous environments. However, their performance under extreme conditions—such as low temperatures, dehydration, and narrow electrochemical stability windows—poses significant challenges that limit their broader application in high-performance energy storage systems.

**Figure 11:**
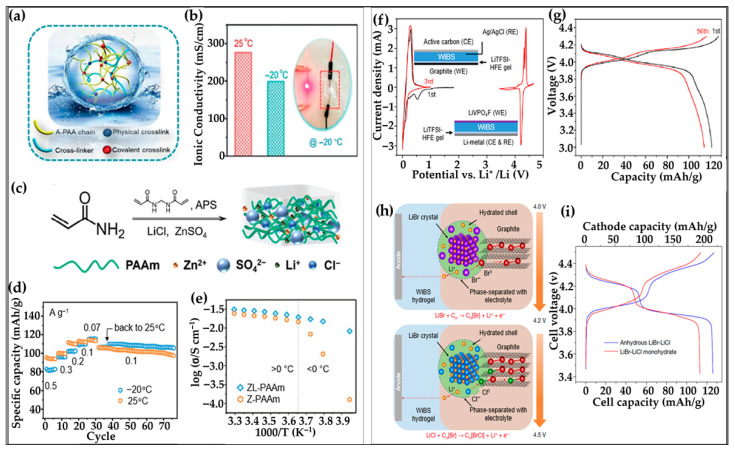
Schematics and experimental data illustrating the design and performance of hydrogel-based electrolytes under varying temperature conditions. (**a**,**b**) Structure and ionic conductivity of KOH-filled A-PAA hydrogel, including its functionality at −20 °C, adapted with permission from ref. [299], Wiley & Sons. (**c**–**e**) Synthesis and electrochemical behavior of ZL-PAAm hydrogel, highlighting rate capability and temperature-dependent conductivity, adapted with permission from ref. [300], Wiley & Sons. (**f**,**g**) Electrochemical characterization of LiTFSI-HFE gel-coated electrodes in hydrogel-WiBS systems, including cyclic voltammetry and voltage profiles, adapted with permission from ref. [239], Elsevier. (**h**,**i**) Conversion–intercalation mechanism and voltage profiles of LBC-G composite cathodes in WiBS-based full cells with different lithium salt hydrates, adapted with permission from ref. [306], Springer Nature.

One of the primary limitations is the behavior of water within hydrogel matrices. At temperatures below 0 °C, water in the electrolyte freezes, drastically reducing solvating ability and ionic mobility. This leads to salt crystallization and loss of structural flexibility, severely impairing battery performance. The freezing behavior is governed by the interaction energy between water molecules (E₍ww₎ ≈ −5.75 kcal mol^−1^). In conventional PVA hydrogels, the polymer–water interaction energy (E₍pw₎ ≈ −6.11 kcal mol^−1^) is only marginally stronger, which is insufficient to prevent freezing. In contrast, PAA hydrogels, due to their highly polarized carboxylic groups (–COOH), exhibit a much stronger interaction with water (E₍pw₎ ≈ −12.92 kcal mol^−1^), effectively disrupting ice formation. As a result, PAA-based hydrogel electrolytes maintain high conductivity (20 × 10^−2^ S cm^−1^) even at −20 °C (Figure 11a,b) [299].

To further enhance low-temperature performance, Zhu et al. [300] developed a dual-salt hydrogel system by incorporating 2 M ZnSO_4_ and 4 M LiCl into a PAAm matrix (ZL-PAAm). The synergistic effect of strongly hydrated ions (Zn^2^⁺ and SO_4_^2−^) and electrostatic stabilization from Li⁺ and Cl^−^ ions reduced water crystallization. This enabled Zn/LiFePO_4_ batteries to perform reliably at −20 °C, with electrochemical characteristics comparable to those at room temperature (Figure 11c–e).

Another major challenge is dehydration, especially under ambient or elevated temperatures. Water’s high vapor pressure (4.25 kPa at 30 °C) makes hydrogel electrolytes prone to drying out, unlike organic solvents such as ethylene carbonate (7.8 Pa). Gong et al. [233] addressed this by incorporating hygroscopic salts like 12 M LiCl into PAAm hydrogels, which formed a tightly bound solvation sheath around water molecules, significantly reducing evaporation. Similarly, bisalt aqueous electrolytes demonstrated improved dehydration resistance, with hydrate melts showing vapor pressures as low as 0.5 kPa [301]. Additional strategies include coating hydrogels with elastomeric layers to retain moisture [302,303,304].

Hydrogels also face limitations due to their narrow electrochemical stability window (~1.23 V), which restricts their use in high-voltage lithium-ion batteries. While “water-in-salt” electrolytes (e.g., 21 M LiTFSI) can extend this window to ~3.0 V [305], they remain incompatible with typical anode materials like Li metal, graphite, and silicon due to cathodic instability. To overcome this, researchers have developed protective strategies such as coating anodes with highly fluorinated ether (HFE) gels to form stable solid electrolyte interphases (SEIs). This allows hydrogel electrolytes to operate with conventional Li-ion electrodes. For instance, WiBS electrolytes gelled in PVA or PEO showed stable redox behavior with graphite and LiVPO_4_F electrodes (Figure 11f–g) [239]. Yang et al. [306] further demonstrated a high-voltage aqueous battery using WiBS–PEO hydrogel with a graphite anode and a (LiBr)_0.5_–(LiCl)_0.5_–graphite (LBC-G) cathode, achieving ~120 mAh g^−1^ capacity and ~4 V operating voltage (Figure 11h–i).

These studies collectively demonstrate that through strategic material engineering—such as salt selection, polymer backbone design, and interface protection—hydrogels can be adapted to function effectively under extreme conditions. Understanding the molecular interactions between water, ions, and polymer chains is essential for designing robust hydrogel electrolytes for next-generation energy storage technologies.

### 6.2. Hydrogels for Sodium-Ion Batteries

Sodium-ion batteries (NIBs) have gained attention as a promising alternative to lithium-ion batteries due to sodium’s natural abundance, low cost, environmental sustainability, and suitable redox potential (E° Na⁺/Na = −2.71 V) [201,202]. With a theoretical energy density of 1165 Wh kg^−1^, NIBs are well-suited for large-scale energy storage and decarbonization efforts [307,308,309].

Despite these advantages, NIBs face several challenges that hinder their practical deployment. These include low energy density, poor cycling stability, limited low-temperature tolerance, and water-induced parasitic reactions. A major issue is the large ionic radius of Na⁺, which complicates fast ion diffusion and leads to structural degradation and volume changes in electrode materials during cycling. This results in poor reversibility and dissolution of active materials, especially in anodes, reducing their electrochemical contribution [310].

Hydrogels offer a versatile solution to these problems. Their crosslinked polymer networks retain water and improve mechanical and electrochemical stability [311]. The porous structure of hydrogels accommodates volume expansion and facilitates rapid Na⁺ transport during insertion/extraction [312]. Additionally, hydrogels exhibit excellent stretchability and ionic conductivity, making them suitable for electrolyte modification and enhancing compatibility with electrodes. Their responsiveness to stimuli also enables the design of self-adaptive structures for low-temperature operation [313].

**Figure 12:**
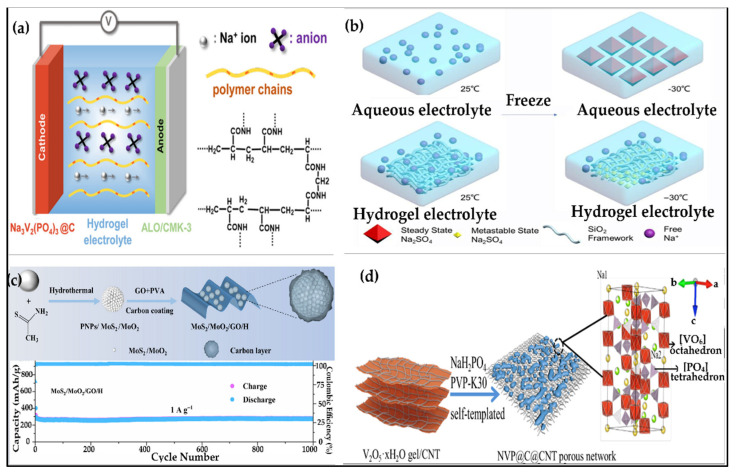
Representative schematics and performance data highlighting the application of hydrogel-based electrolytes in sodium-ion batteries (NIBs). (**a**) Configuration of an NIB employing a polyacrylamide hydrogel electrolyte with ALO/CMK-3 anode and NVP@C cathode, adapted with permission from ref. [311], copyright© 2018 American Chemical Society. (**b**) Comparison of freezing behavior between aqueous Na_2_SO_4_ and Na_2_SO_4_–SiO_2_ hydrogel electrolytes from 25 °C to −30 °C, adapted with permission from ref. [313], Elsevier. (**c**) Preparation and long-term cycling performance of MoS_2_/MoO_2_/GO/H composite anode, adapted with permission from ref. [315], Elsevier. (**d**) Synthesis schematic of Na_3_V_2_(PO_4_)_3_@C@CNT composite cathode, adapted with permission from ref. [312], Elsevier.

Hydrogels are non-toxic, low-cost, and easily modifiable, supporting the development of safer and more sustainable NIBs. Recent designs have incorporated hydrogels to improve thermal stability, mechanical flexibility, and interface contact, while also suppressing electrolyte leakage. In these systems, hydrogels have been applied as electrolytes [311,313,314], anode materials [315], and templates for cathodes [310,312], as illustrated in Figure 12a–d.

#### 6.2.1. Hydrogel Electrolytes for Sodium-Ion Batteries

To address the low-temperature limitations of sodium-ion batteries (NIBs), Cheng et al. [313] developed a Na_2_SO_4_–SiO_2_ hydrogel electrolyte using fumed silica, Na_2_SO_4_, and methyl alcohol (Figure 12b). This design leverages the strong polarity of Na_2_SO_4_ and the anti-freezing properties of methyl alcohol to suppress salt precipitation and maintain ionic conductivity at sub-zero temperatures. The hydrogel forms stable Si–O–Si bridges and hydrogen bonds, enabling a conductivity of 0.070 mS cm^−^^1^ at −30 °C. The resulting NIB delivered reversible capacities of 78.9 mAh g^−1^ at −20 °C and 61.8 mAh g^−1^ at −30 °C, demonstrating excellent low-temperature performance. Zhong et al. [311] tackled the issue of anode dissolution by pairing an organic alloxazine (ALO) anode with a conductive carbon material (CMK-3) and a polyacrylamide hydrogel electrolyte. The hydrogel preserved water content and stabilized the interface, while CMK-3 enhanced conductivity and reduced ALO dissolution. The optimized system achieved a high capacity of 160 mAh g^−^^1^, an average discharge voltage of 1.03 V, and an energy density of 50 Wh kg^−^^1^, with 90% capacity retention after 100 cycles at 2 C and 146 mAh g^−^^1^ at 10 C.

#### 6.2.2. Hydrogel Anodes for Sodium-Ion Batteries

To improve anode performance and cycling stability, Zhang et al. [315] developed a 3D MoS_2_/MoO_2_/graphene oxide/polyvinyl alcohol hydrogel composite using a cationic absorption method, hydrothermal synthesis, and gelation (Figure 12c). MoS_2_ offers high capacity and a layered structure but suffers from poor conductivity and volume expansion. MoO_2_ was introduced to form a stable heterojunction, while graphene oxide provided mechanical strength and conductivity. The resulting hydrogel composite formed a highly conductive, ultrathin carbon network with excellent electrochemical kinetics. The anode delivered 350 mAh g^−1^ after 100 cycles at 0.1 A g^−1^ and 273 mAh g^−1^ after 1000 cycles at 1 A g^−1^, demonstrating outstanding long-term cycling stability and minimal capacity decay.

#### 6.2.3. Hydrogel Cathodes for Sodium-Ion Batteries

To improve the performance and cycling stability of sodium-ion battery (NIB) cathodes, researchers have explored hydrogel-based porous network designs and surface engineering strategies. These approaches aim to address challenges such as limited ion transport, poor conductivity, and structural degradation during cycling. Yan et al. [312] developed a hydrogel self-templated method to fabricate a 2D porous Na_3_V_2_(PO_4_)_3_@C@CNT (NVP) cathode structure (Figure 12d). The design integrates 0D carbon-coated Na_3_V_2_(PO_4_)_3_ nanoparticles with 1D carbon nanotubes (CNTs), forming a conductive and open network that facilitates rapid Na⁺ diffusion and accommodates volume changes. The carbon coating enhances electronic conductivity and interparticle contact, while the porous architecture supports long-term structural integrity. As a result, the NVP cathode exhibits exceptional cycling stability—retaining 94% of its capacity after 4500 cycles at 5 C—and delivers a high rate capability of 84 mAh g^−1^ at 10 C. Complementing this, Cheng et al. [310] synthesized a 3D porous Na_3_V_2_(PO_4_)/C cathode using hydrogel and hydrothermal methods. The resulting particles (100–200 nm) offer high surface area and conductivity. This cathode achieved a specific capacity of 62.1 mAh g^−1^ at 10 C, with 98% retention after 140 cycles at 1 C. Even after 2000 cycles at 5 C, the capacity remained at 80.4 mAh g^−1^, with 86% retention.

These studies demonstrate the effectiveness of hydrogel-based cathode designs in enhancing the electrochemical performance, rate capability, and long-term durability of NIBs, paving the way for more robust and scalable energy storage technologies.

### 6.3. Hydrogel Electrolytes for Zinc-Ion Battery

**Figure 13:**
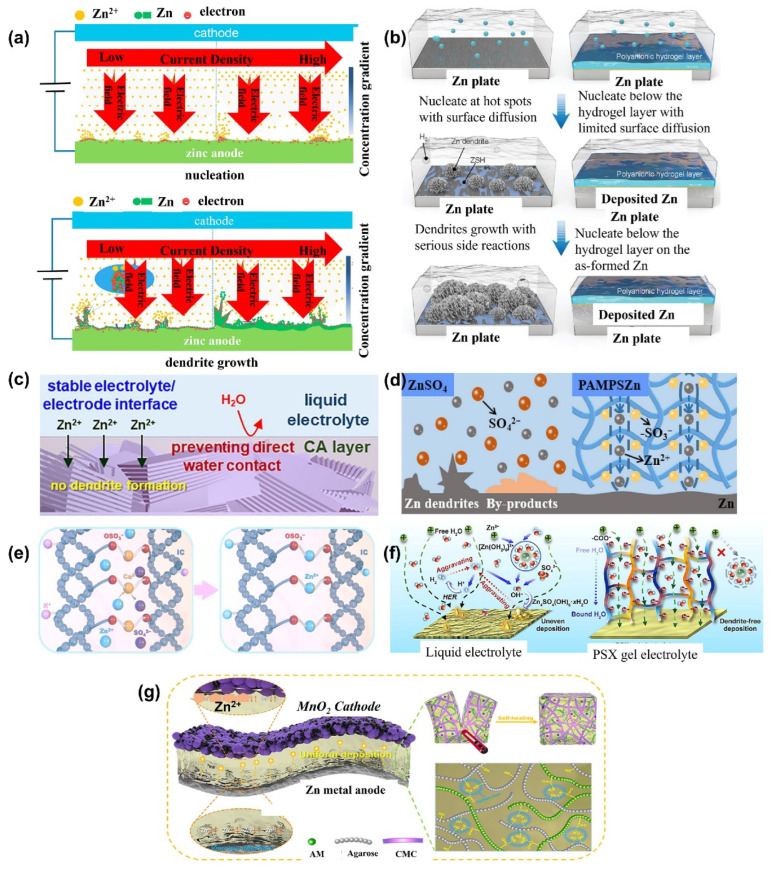
Schematics and illustrations highlighting recent strategies to address zinc dendrite formation and enhance electrolyte stability in zinc-based batteries. (**a**) Mechanistic depiction of zinc dendrite growth, redrawn and reproduced with permission [318], Elsevier. (**b**) comparison of Zn deposition behavior on bare Zn and Zn–SHn surfaces, redrawn and reproduced with permission [329], Wiley & Sons. (**c**) influence of stable electrolyte formulations on dendrite suppression, reproduced with permission from ref. [324], Elsevier. (**d**) polymer chains containing –SO_3_^−^ groups to regulate Zn^2^⁺ migration, reproduced with permission from ref. [325], Elsevier. (**e**) design of single zinc-ion conducting hydrogel electrolytes, reproduced with permission [331], copyright© 2021 American Chemical Society.; (**f**) self-healing hydrogel electrolyte systems, reproduced with permission from ref. [332], Elsevier; and (**g**) hydrogel networks incorporating polymeric anions for improved ion transport and structural integrity, reproduced with permission from ref. [333], Elsevier.

Hydrogel electrolytes have emerged as a promising solution to address key challenges in zinc-ion batteries (ZIBs), which are increasingly favored for their safety, affordability, and environmental compatibility [32,33,316,317]. Despite these advantages, ZIBs face significant limitations, including zinc dendrite formation, side reactions, and a narrow electrochemical stability window due to water decomposition. Dendrites, which form through uneven Zn^2^⁺ deposition during cycling, can penetrate the separator and cause internal short circuits, posing serious safety risks [318]. The probability of such failures increases under high current densities and is exacerbated by surface defects and polarization effects. Additionally, side reactions at the electrode–electrolyte interface consume active materials and generate gases such as H_2_ and O_2_, leading to capacity loss and reduced Coulombic efficiency [319,320]. As illustrated in Figure 13a, [318] dendrite nucleation and growth are governed by epitaxial and spiral dislocation mechanisms, influenced by factors such as surface tension and temperature gradients [321,322]. Hydrogel electrolytes mitigate these issues through their interconnected porous networks that facilitate uniform ion transport [323], functional groups (–OH, –COOH, –NH_2_) that coordinate with Zn^2^⁺ to suppress hydrogen evolution [30], and mechanical robustness that restrains dendrite growth. Moreover, their self-healing properties and thermal stability enhance long-term performance and enable operation under extreme conditions. By stabilizing the electrode interface and extending the voltage window, hydrogels significantly improve the safety, cycling life, and energy density of ZIBs, making them a critical component in the advancement of next-generation aqueous battery technologies.

To overcome the electrochemical and mechanical limitations of zinc-ion batteries (ZIBs), researchers have developed multifunctional hydrogel electrolytes with tailored properties that enhance performance, safety, and longevity. Key design strategies include engineering compatible electrode–electrolyte interfaces, incorporating additives such as plasticizers and metal ions, and introducing anionic chains to regulate Zn^2^⁺ transport [324,325,326,327,328]. These approaches have led to the development of hydrogels with enhanced mechanical strength, thermal stability, and self-healing capabilities, which effectively suppress dendrite growth and mitigate side reactions. For instance, Yang et al. [329] designed a zincophilic polyanionic hydrogel chemically bonded to the Zn anode via O–Zn linkages, improving interfacial adhesion and suppressing hydrogen evolution (Figure 13b). Park et al. [324] utilized a natural polysaccharide-based hydrogel to form a biocompatible barrier that reduced corrosion and enabled long-term cycling (Figure 13c). Functional composites such as PAM/phosphonated graphene oxide [326], Na-montmorillonite/PAM hybrids [327], and dual-network PAMPS/PAM hydrogels [328] have demonstrated improved ionic conductivity and mechanical integrity. Anion chain-directed strategies further enhence Zn^2^⁺ transport, as shown by polyanionic hydrogels [328], polyzwitterionic systems [330], and single-ion electrolytes [331].

Cong et al. [325] synthesized a polyanionic hydrogel via ion exchange and radical polymerization, guiding Zn^2^⁺ along confined pathways and reducing side reactions (Figure 13d). Chan et al. [331] developed a single-ion Zn^2^⁺ hydrogel electrolyte (P(ICZn-Aam)) using iota carrageenan and acrylamide. The SO_3_^2−^ groups facilitate Zn^2^⁺ migration along the polymer chain, enhancing ion transport and interfacial stability, leading to improved Zn deposition and cycling performance (Figure 13e). Fu et al. [332] achieved high ionic conductivity and Zn^2^⁺ migration number using a PVA–xanthan gum hydrogel (Figure 13f). Ling et al. [333] incorporated carboxymethyl cellulose (CMC) into agarose and polyacrylamide (PAM) networks, forming a porous, hydrophilic structure. This enhanced ionic conductivity, achieving 23.1 mS·cm^−1^ at 25 °C (Figure 13g). Collectively, these innovations in hydrogel design offer a versatile platform for addressing dendrite formation and side reactions, paving the way for high-performance, durable ZIBs.

#### 6.3.1. High-Voltage Hydrogel-Based Zinc-Ion Batteries

Achieving high operating voltage in zinc-ion batteries (ZIBs) is crucial for enhancing energy density and meeting the demands of advanced energy storage systems. However, aqueous ZIBs are inherently limited by parasitic reactions such as hydrogen evolution (HER) and oxygen evolution (OER), triggered by water decomposition beyond its electrochemical stability window (~1.23 V). These side reactions reduce Coulombic efficiency, accelerate electrode degradation, and promote dendrite formation. To address these challenges, researchers have employed strategies including reducing free water content, introducing highly concentrated zinc salts, incorporating anion chains for guided ion transport, and engineering stable electrode–electrolyte interfaces [334,335,336]. Hydrogel electrolytes have proven effective in this context due to their ability to bind water molecules and suppress side reactions, enabling stable high-voltage operation.

**Figure 14:**
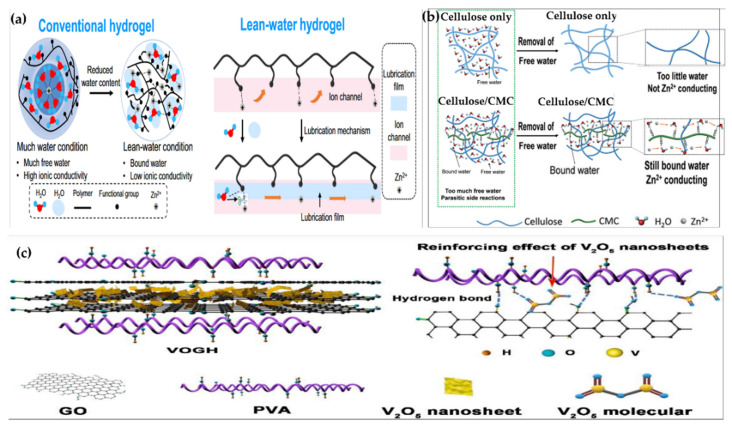
Schematics illustrating advanced electrolyte strategies in zinc-ion batteries: (**a**) lean-water electrolyte systems designed to minimize side reactions [334]; (**b**) bound-water electrolytes enhancing stability under low-temperature conditions [335]; and (**c**) engineered interfaces promoting hydrogen bonding between the electrolyte and electrode for improved compatibility and performance, adapted with permission from ref. [338], Elsevier.

For example, Wang et al. [334] developed a water-poor polyzwitterionic hydrogel with –SO_3_^2−^ and –C_3_N⁺ groups, achieving a voltage window exceeding 2 V and ionic conductivity of 2.6 × 10^−^^3^ S cm^−1^ (Figure 14a). Xu et al. [335] developed a cellulose-based hydrogel electrolyte using 2 wt% cellulose, 0.1 wt% N,N′-methylenebisacrylamide (MBA) as the crosslinking agent, and 0.2 wt% ammonium persulfate (APS) as the initiator. The hydrogel was subjected to a freeze-drying process at –20 °C for 12 h to enhance its mechanical integrity and reduce free water content (Figure 14b). This design suppresses dendrite growth and minimizes free water content, reducing side reactions at high voltage. As a result, it enables stable, high-power energy storage under elevated voltage and current conditions.

Interface engineering has also played a vital role, as demonstrated by Ma et al. [336] using sol–gel modified hydrogels with Prussian Blue cathodes, and by Wu et al. [337] and Lv et al. [338] employing freeze–thaw cycles to enhance hydrogen bonding and interfacial stability (Figure 14c).

Xu et al. [335] and Li et al. [339] fabricated cellulose-based hydrogels with bound water and strong hydrogen bonding, achieving voltage windows up to 1.9 V and energy densities of 255.4 Wh kg^−1^. 

**Figure 15:**
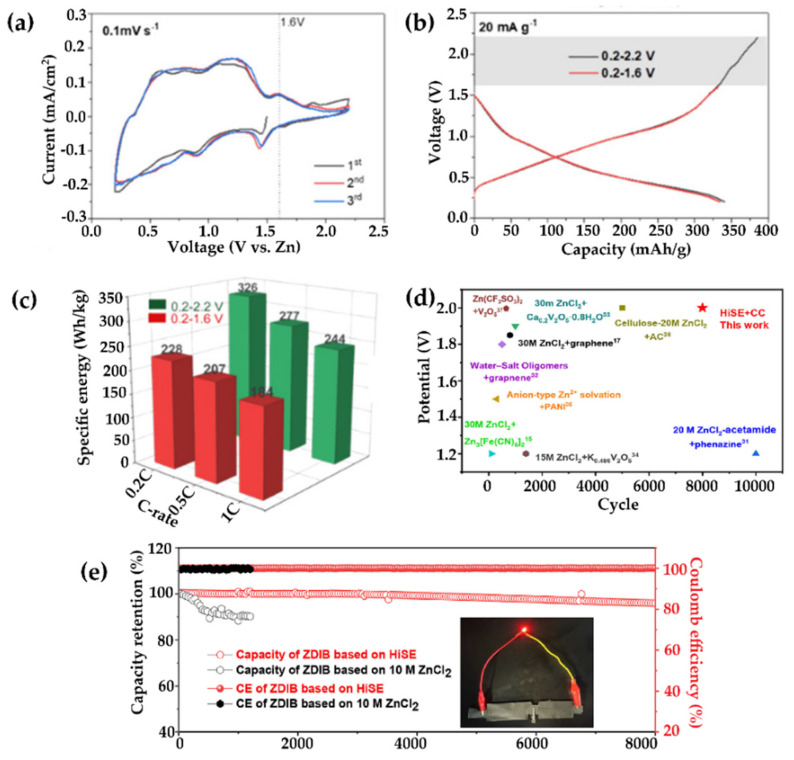
Electrochemical performance and voltage behavior of zinc-ion batteries using gel polymer and hydrogel-based electrolytes. (**a**) Cyclic voltammogram of Zn/V_2_O_5_ cells with gel polymer aqueous electrolyte; (**b**) corresponding charge–discharge profiles; (**c**) specific energy across different voltage windows, adapted with permission [342] copyright© 2020 American Chemical Society. (**d**) Comparative analysis of potential windows for zinc-ion batteries with different electrolytes systems; (**e**) cycling stability of hydrogel water-in-salt electrolyte versus 10 M ZnCl_2_ electrolyte, adapted with permission from ref. [343], Elsevier.

Zhang et al. [340] and Lu et al. [341] introduced multi-crosslinked and biodegradable hydrogels, respectively, both demonstrating stable cycling and voltage ranges up to 2.0 V. Additionally, Zhang et al. [342] and Sun et al. [343] innovations include gelatin-based hydrogels, PVA-based systems doped with LiTFSI and ZnOTf_2_ (Figure 15a–e), and PVA–gelatin hydrogels with high ZnCl_2_ concentration, all of which convert free water into bound water and suppress side reactions.

#### 6.3.2. Self-Healing Hydrogel-Based Zn-Ion Batteries

**Figure 16:**
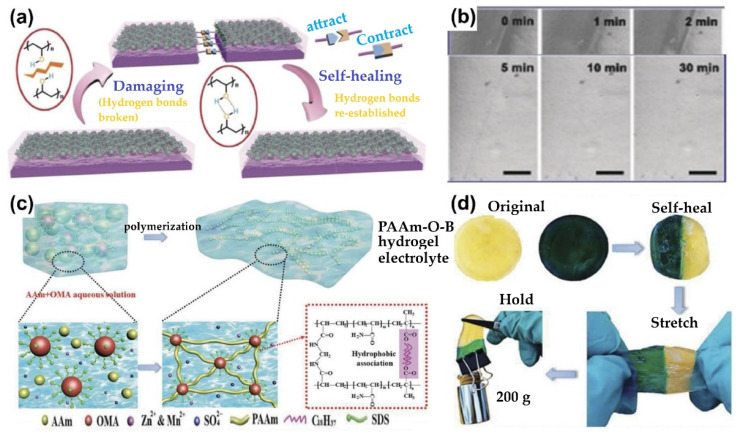
Schematics and visual demonstrations of self-healing hydrogel electrolytes in zinc-ion batteries. (**a**,**b**) Design and healing behavior of an integrated all-in-one self-healing ZIB system, redrawn and reproduced with permission [345], Wiley & Sons; (**c**) synthesis pathway and (**d**) self-repairing capability of PAAm-O-B hydrogel electrolyte, adapted with permission [346], Wiley & Sons.

In the context of wearable and flexible electronics, zinc-ion batteries (ZIBs) are frequently subjected to mechanical stress such as bending, twisting, and stretching, which can lead to electrolyte leakage, structural fracture, and diminished ionic conductivity. These mechanical deformations compromise electrochemical performance and pose safety risks, especially under repeated fatigue that disrupts the conductive network and shortens battery lifespan. To address these challenges, self-healing hydrogel electrolytes have been developed, leveraging dynamic and reversible interactions—including covalent and non-covalent bonding, ionic crosslinking, hydrogen bonding, and host–guest interactions—to autonomously restore mechanical integrity and electrochemical functionality [344]. Huang et al. [345] demonstrated a PVA/Zn(TFSI)_2_ hydrogel fabricated via freeze–thaw cycles, where –OH groups enabled hydrogen bonding and self-healing after multiple cuts (Figure 16a,b).

Liu et al. [347] constructed a flexible ZIB using VS_2_ nanosheets and Zn nanowires with a PVA-based self-healing electrolyte, achieving high capacity and mechanical resilience. Li et al. [348] introduced a COO^−^–Fe-modified PVA hydrogel in a quasi-solid-state Zn/MnO_2_ battery, which not only self-healed but also suppressed dendrite growth and side reactions. Liu et al. [349] incorporated Hbimcp into PPO chains, forming Zn^2^⁺-ligand complexes with reversible coordination bonds that enabled dynamic repair and high ionic conductivity. Additional innovations include zinc alginate hydrogels with carboxylate groups [350], double-crosslinked PAM hydrogels capable of enduring 50 fracture/healing cycles [346] (Figure 16c,d), and PAM-based Zn/Mn-doped hydrogels fabricated via photoinitiated polymerization [225]. Liu et al. [351] enhanced safety and reversibility using Zn^2^⁺-induced supramolecular interactions in chitosan/PAM hydrogels. To further improve mechanical strength, Shen et al. [352] embedded halloysite nanotubes (HNTs) into PAM networks, resulting in superior dissipation and hydrogen bonding. These self-healing hydrogel systems collectively offer robust mechanical adaptability and electrochemical stability, making them ideal for next-generation flexible and wearable ZIB applications.

### 6.4. Hydrogels for Magnesium-Ion Batteries

Magnesium-ion batteries (MIBs) are gaining attention as sustainable alternatives to lithium-ion batteries due to magnesium’s abundance, low cost, non-toxicity, high volumetric capacity (3833 mAh cm^−3^), and dendrite-free cycling [353,354,355]. Magnesium is also lightweight (1.74 g cm^−3^) and stable in ambient conditions, making it suitable for use as a pure metal anode.

However, MIBs face several challenges that hinder their practical deployment. The primary issue lies in the incompatibility between magnesium anodes and conventional electrolytes. Magnesium readily forms a passivation layer in contact with common solvents (e.g., esters, carbonates, nitriles) and contaminants (e.g., water, CO_2_), which blocks Mg^2^⁺ transport and impairs reversibility [356]. Additionally, MIBs suffer from limited oxidative stability, sluggish Mg^2^⁺ diffusion in cathodes due to strong electrostatic interactions, and insufficient energy density and cycle life [357,358].

To address these limitations, research has focused on optimizing MIB components—anodes, cathodes, electrolytes, and interfacial materials [357,358,359]. Hydrogels have emerged as promising candidates due to their tunable structure, low toxicity, and ability to support Mg^2^⁺ mobility. Their porous networks and stimuli-responsive properties can enhance ionic conductivity and suppress uneven Mg deposition, even under extreme conditions [359,360,361].

#### 6.4.1. Hydrogels as Electrolyte for Mg-Ion Battery (MIBs)

**Figure 17:**
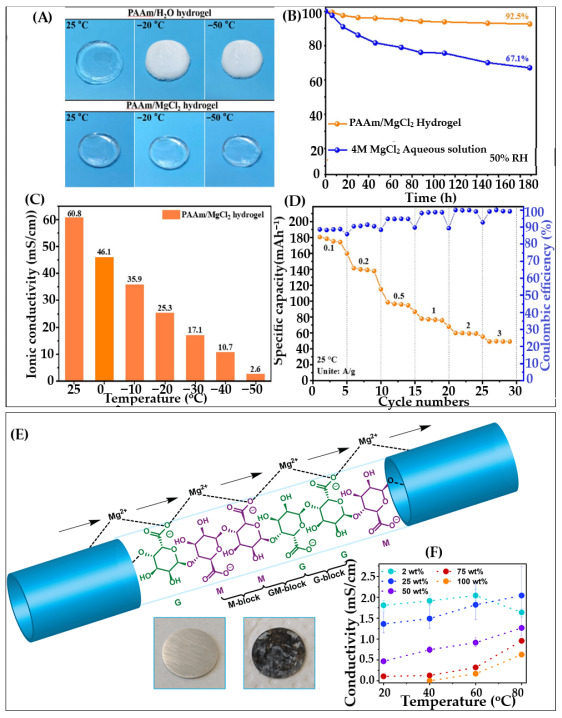
Multifunctional roles of hydrogels in magnesium-ion batteries (MIBs). (**A**) photographs of PAAm/H_2_O and PAAm/MgCl_2_ hydrogel electrolytes at various temperatures (25 °C, −20 °C, −50 °C); (**B**) mass change under 50% relative humidity at room temperature; (**C**) ionic conductivity across a temperature range from −50 °C to +25 °C; and (**D**) specific capacity of quasi-solid-state MIBs at 25 °C; Adapted with permission from [360], Elsevier. (**E**) Schematic overview of Mg^2+^ ‘hopping’ between carboxylates (**F**) Conductivity of electrolytes at different concentrations. [362] MDPI.

Despite their potential, hydrogel applications in MIBs remain in early stages. Yang et al. [360] pioneered the use of hydrogels as electrolytes for MIBs (Figure 17A–D), targeting performance degradation at sub-zero temperatures. They introduced an unconventional deep eutectic solvent (DES)-based hydrogel using polyacrylamide (PAAm) and MgCl_2_. Unlike conventional DESs, which suffer from high viscosity and low conductivity at low temperatures, the MgCl_2_-based DES disrupts hydrogen bonding among water molecules, lowering the freezing point and enhancing ionic mobility. The resulting PAAm/MgCl_2_ hydrogel electrolyte demonstrated exceptional anti-freezing properties (freezing point: −62 °C) and high ionic conductivity (2.77 mS cm^−1^ at −50 °C). The assembled quasi-solid-state MIB exhibited stable electrochemical performance across a wide temperature range (+25 to −50 °C), with a discharge capacity of 97.9 mAh g^−1^ at 0.1 A g^−1^ and a high cycling stability of 1000 cycles. It retained 28% of its specific capacity at 3 A g^−1^, confirming its robustness under harsh conditions.

#### 6.4.2. Hydrogel-Derived Anodes for MIBs

To develop high-performance anodes for MIBs, Cheng et al. [361] proposed a strategy to mitigate the mechanical and structural degradation typically caused by volume fluctuations during magnesiation/demagnesiation. These fluctuations often lead to agglomeration and pulverization of the anode material, compromising battery performance. To address this, the researchers encapsulated bismuth (Bi) nanoparticles within a cellulose nanocrystal (CNC) hydrogel-derived carbon aerogel matrix. The carbon substrate acts as a mechanical buffer, accommodating volume changes, while its 3D porous structure facilitates efficient electron and ion transport. Moreover, the uniform dispersion of Bi nanoparticles within the carbon network prevents agglomeration and enhances structural integrity.

Hydrogels have emerged as a promising class of materials for solid-state and quasi-solid-state electrolytes due to their high water content, tunable ionic conductivity, and mechanical flexibility. In the context of magnesium-ion batteries (MIBs), alginate-based hydrogels offer a sustainable and biocompatible platform for electrolyte design. The study on magnesium alginate electrolytes by Markus et al. [362] demonstrates how Mg^2^⁺ ions serve a dual function—acting both as ionic charge carriers and as crosslinking agents that stabilize the hydrogel network (Figure 17E). The alginate hydrogel is formed through ionic crosslinking between guluronic acid blocks in the alginate chains and divalent Mg^2^⁺ ions, resulting in a three-dimensional polymer matrix with enhanced mechanical integrity. This structure not only supports efficient Mg^2^⁺ ion transport but also provides dimensional stability and leakage resistance, which are critical for safe and durable battery operation.

Electrochemical evaluations revealed that the magnesium alginate hydrogel supports stable cycling performance, with consistent ionic conductivity suitable for room-temperature applications. The hydrogel’s quasi-solid-state nature also contributes to improved safety, flexibility, and environmental compatibility, making it a viable candidate for next-generation green and wearable energy storage systems.

In summary, the magnesium alginate hydrogel plays a central role in enabling a mechanically robust, ionically conductive, and eco-friendly electrolyte system for MIBs, bridging the gap between performance and sustainability in solid-state battery technologies.

### 6.5. Hydrogels for Aluminum-Ion Batteries

Aluminum-ion batteries (AIBs) have emerged as a compelling alternative to lithium-ion batteries due to aluminum’s natural abundance, low cost, and safety profile. Aluminum offers a trivalent charge carrier (Al^3^⁺), which contributes to a high theoretical gravimetric capacity of 2980 mAh g^−1^ and an exceptional volumetric capacity of 8040 mAh cm^−3^. AIBs typically consist of an Al-based anode, a cathode capable of hosting Al^3^⁺ ions, an electrolyte, binder, and current collector. During discharge, Al atoms at the anode oxidize to release electrons and form Al^3^⁺ ions, which migrate through the electrolyte to the cathode. The reverse occurs during charging.

AIBs are generally categorized into aqueous and non-aqueous systems based on the electrolyte solvent. Aqueous AIBs are environmentally friendly and cost-effective but face significant challenges: hydrogen evolution occurs before effective Al^3^⁺ reduction, aluminum corrosion leads to passivating oxide films, and electrolyte decomposition impedes ion transport. Consequently, research has shifted toward non-aqueous AIBs, which offer better electrochemical stability, higher safety (non-volatility and non-flammability), and a broader electrochemical window.

Despite these advantages, AIBs still face fundamental limitations. The relatively high redox potential of aluminum (−1.66 V) compared to lithium (−3.04 V) and sodium (−2.71 V) results in a lower working voltage. Additionally, the trivalent nature of Al^3^⁺ leads to sluggish ion kinetics, high overpotentials, and structural collapse of host materials during cycling [357]. Electrolyte leakage, unstable interfaces due to mechanical deformation, and poor separator compatibility further hinder performance. Moreover, many polymers effective in lithium-ion batteries fail in AIBs due to their inability to transport Al^3^⁺ or their incompatibility with Lewis acidic ionic liquids used in non-aqueous systems.

Hydrogels have gained attention as potential electrolytes for AIBs due to their high ionic conductivity, chemical and thermal stability, non-toxicity, and low cost. Their abundant functional groups can form strong interactions with Al^3^⁺, enhancing mechanical strength and crosslinking. However, hydrogel applications in AIBs are still in early development, and careful materials selection is crucial to ensure compatibility with aluminum chemistry.

#### Hydrogels as Electrolytes for AIBs

**Figure 18:**
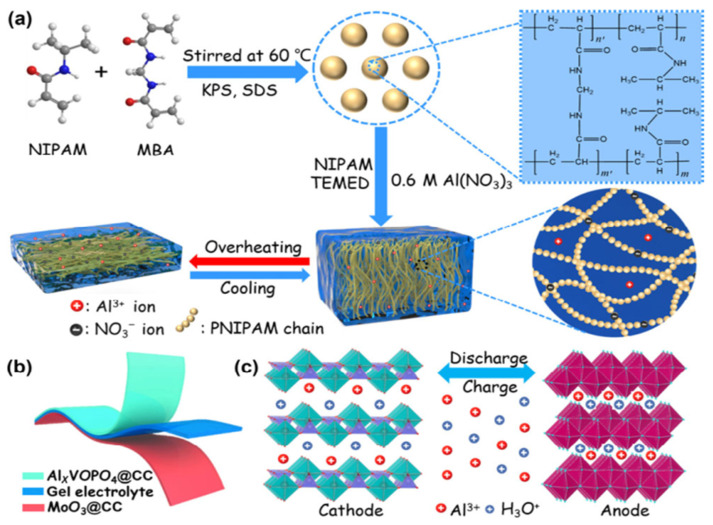
Design and functional integration of hydrogel-based smart electrolytes in hybrid ion batteries (HIBs). (**a**) Schematic of the synthesis route for PNIPAM hydrogel-based smart electrolyte; (**b**) configuration of a flexible aqueous HIB comprising AlXVOPO_4_·2H_2_O@carbon cloth cathode, PNIPAM hydrogel electrolyte, and MoO_3_@carbon cloth anode; (**c**) working principle of the HIB system based on intercalation-type electrodes, adapted with permission [363], Springer Nature.

To address the need for stable and aluminum-compatible electrolytes, Wang et al. [363] developed a thermoresponsive hydrogel electrolyte based on poly(N-isopropylacrylamide) (PNIPAM) (Figure 18).

Synthesized via free radical polymerization, PNIPAM features a balanced structure of hydrophilic amide (-CONH) and hydrophobic isopropyl (-CH(CH_3_)_2_) groups. At ambient temperature (24 °C), hydrogen bonding between water molecules and -CONH groups expands the hydrogel network, creating micropores that facilitate Al^3+^ migration and enhance ionic conductivity (1.8 S/m). As temperature rises, the hydrogel network contracts due to weakened hydrogen bonds and strengthened hydrophobic interactions, reducing conductivity and preventing thermal runaway. The assembled battery—Al_x_VOPO_4_·2H_2_O cathode//PNIPAM hydrogel electrolyte//MoO_3_ anode—exhibited a high specific capacity of 125 mAh g^−1^ at 0.1 A g^-1^ and remarkable cycling stability over 10,000 cycles at 2 A g^−1^, with no capacity fading.

**Figure 19:**
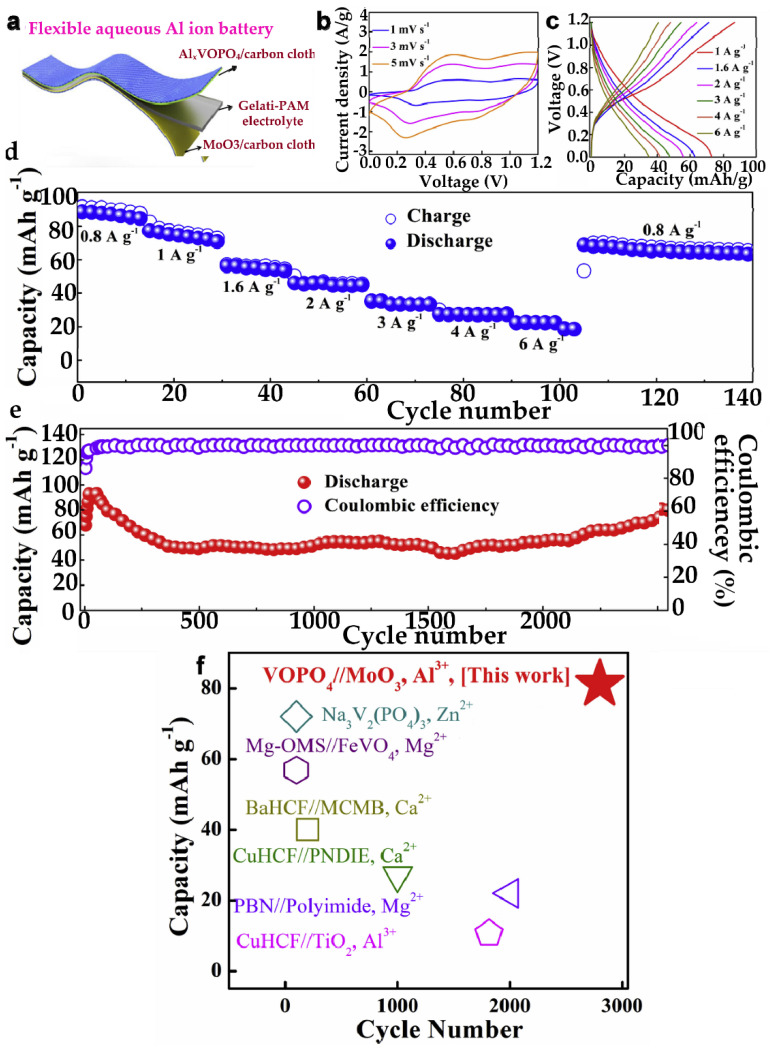
Electrochemical performance metrics of a rechargeable solid-state aqueous aluminum-ion battery (AIB). (**a**) Structural schematic of the flexible AIB design; (**b**) cyclic voltammetry profiles at varying scan rates; (**c**) charge–discharge curves under different current densities; (**d**) rate capability across multiple operating conditions; (**e**) long-term cycling stability and coulombic efficiency at 1 A g^−1^; and (**f**) comparative performance analysis of the presented AIB with other aqueous multivalent metal-ion battery systems, adapted with permission [364], Elsevier.

In another study, Wang et al. [364] employed a gelatin–polyacrylamide hydrogel electrolyte to fabricate a safe and flexible AIB (Figure 19).

The battery used MoO_3_ and VOPO_4_ as intercalation electrodes. This design achieved a high rate capability (6 A g^−1^), discharge capacity of 88 mAh g^−1^, and long-term stability with 86.2% capacity retention after 2800 cycles. Notably, the battery demonstrated practical Al storage capabilities by powering electroluminescent panels of 1 m and 100 cm^2^, highlighting its potential for real-world applications.

**Figure 20:**
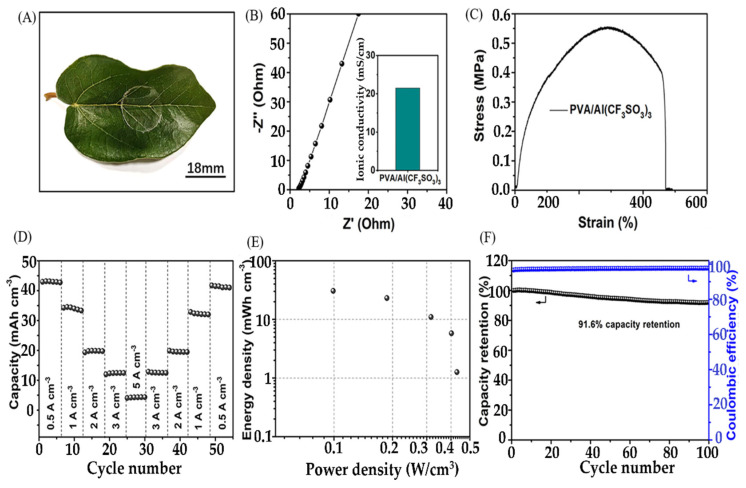
Electrochemical and mechanical characterization of PVA/Al(CF_3_SO_3_)_3_ hydrogel electrolyte for aluminum-ion batteries. (**A**) Optical image of the hydrogel material; (**B**) AC impedance spectrum with inset showing ionic conductivity; (**C**) stress–strain behavior indicating mechanical robustness; (**D**,**E**) rate capability and cycling stability of the battery system; (**F**) long-term cycling performance of Al-ion cells using MnHCF cathode and GO/MoO_3_ anode with hydrogel electrolyte [365].

To suppress alumina passivation on the aluminum anode, Xiong et al. [365] developed a PVA-based hydrogel electrolyte (PVA/Al(CF_3_SO_3_)_3_) (Figure 20).

PVA was selected for its excellent mechanical properties, high water absorbency, low cost, and non-toxic thermoplastic nature. The team fabricated a stretchable fiber-shaped AIB using a Mn-hexacyanoferrate cathode, a graphene oxide-decorated MoO_3_ anode, and the PVA-based hydrogel. The electrolyte exhibited high ionic conductivity (21.6 mS cm^−1^), tensile modulus of 0.55 MPa, and strain of 461%, enabling excellent flexibility. The battery delivered a specific capacity of 42 mAh cm^−3^ at 0.5 A cm^−3^, retained 91.6% capacity over 100 cycles, and achieved a specific energy of 30.6 mWh cm^−3^. When integrated into wearable textiles, it successfully powered an LED, demonstrating its potential for flexible and wearable electronics [363].

Furthermore, to better understand the volume retention behavior observed in hydrogel-based systems, we analyzed potential failure mechanisms contributing to volume attenuation during extended cycling. One key factor is gel dehydration, which occurs due to gradual water loss from the hydrogel matrix under repeated electrochemical stress and ambient conditions. This dehydration can lead to shrinkage, reduced ionic conductivity, and compromised mechanical integrity [233].

**Figure 21:**
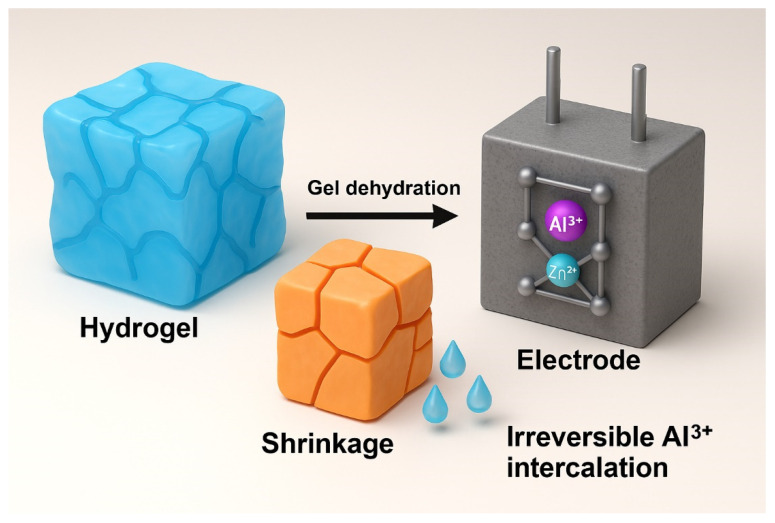
Schematic illustration of volume attenuation mechanisms in hydrogel-based zinc-ion batteries, illustrating gel dehydration-induced shrinkage and irreversible Al^3^⁺ intercalation into the electrode structure.

Additionally, irreversible intercalation of multivalent ions such as Al^3^⁺ into the electrode structure may cause lattice distortion and hinder reversible ion transport, thereby contributing to structural instability and volume loss (Figure 21) [366]. These phenomena collectively impact the long-term dimensional stability and electrochemical performance of the system, despite the observed volume retention rate exceeding 80% after 1000 cycles.

Table 3 summarizes the current strategies adopted to enhance metal-ion battery performance, focusing on modifications to the hydrogel’s porous network and coatings with highly conductive materials.

**Table 3:** Electrochemical and Mechanical Properties of Electrode-Electrolyte Systems with Battery Type.

Battery Type	Electrodes	Electrolyte	Ionic Conductivity	Specific Capacity	Cyclability	Tensile Strength	Elastic Modulus	Maximum Strain	Capacity Retention Under Deformation	Ref.
Li-ion	Anode: prelithiated V_2_O_5_, cathode: LiMn_2_O_4_	PAM-WiS gel	103 S cm^−1^	60 mAh/g at 1C/40 mAh/g at 5C	95% after 100 cycles	—	—	100%	28 mAh/g under 50% stretch	[367]
Li-ion	Anode: activated carbon, cathode: LiMn_2_O_4_	PAAm–chitosan WiS gel	51.3 mS cm^−1^	110.7 mAh/g at 0.1 A/g	90.1% after 2000 cycles	—	—	2540%	~95% under 90° bending and twisting	[368]
Li-ion	Anode: SiO microparticles, cathode: lithium foil	TA–PAA cobweb-like hydrogel binder	—	901.2 mAh/g after 1200 cycles at 2 A/g	619.2 mAh/g at 5 A/g (rate performance)	High (via nanoindentation)	High (via rheology)	Up to 1000%	Maintains structure; self-healing; minimal volume expansion	[281]
Li-ion	Anode: SiP_2_/C composite, cathode: lithium foil	Li-PAA@PEDOT:PSS hydrogel binder	—	1520 mAh/g at 2000 mA/g	>450 cycles, ICE >93.8%	—	—	—	Self-healing and structural recombination	[277]
Li–S	Cathode: 3D carbon-HKUST-1/S, Anode: lithium metal	Conducting polymer hydrogel with MOF domains	—	>16 mAh/cm^2^; 1230.8 mAh/cm^3^	82% after 300 cycles at 0.2C	—	—	—	Maintains structural integrity under high sulfur loading	[284]
Li-ion	Anode: Si nanoparticles, cathode: lithium foil	3D meshlike PAM hydrogel binder	—	≈1526 mAh/g	Stable over 500 cycles	—	Tunable via crosslinker	—	Maintains structure under deformation	[369]
Li-ion	Anode: Si particles, cathode: lithium foil	ESVCA hydrogel binder	—	~1743 mAh/g after 200 cycles	74.1% retention	—	—	—	Self-healing and stretchable up to 400%	[370]
Li-ion	Cathode: standard LIB cathode, Anode: lithium metal	BN/BC composite aerogel separator	—	146.5 mAh/g	500 cycles, 0.012% degradation/cycle	High (BN aerogel)	—	—	Flame-retardant, flexible, wettable	[259]
Li-ion	Cathode: high-loading LIB cathode, Anode: lithium foil	Low-water-content hydrogel film (6.63 wt%)	2.6 mS/cm	119 mAh/g (2.5 mAh/cm^2^ areal)	81.8% after 500 cycles at RT; 66.2% at −30 °C	—	—	High (foldable)	Anti-freezing, low swelling, flexible	[371]
Li-ion	Cathode: stretchable nanowire network	Crosslinked hydrogel	—	119 mAh/g	91.6% after 250 tensile strains	—	—	100% stretchable	Stable under repeated deformation	[291]
Li-ion	Full cell (flexible LIB)	Moisture self-absorbing hydrogel film (4.62% water)	1.15 mS/cm	—	73.2% after 1000 cycles at 1 A/g	—	—	>300%	Maintains OCV under 50–125% stretch and after cutting	[230]
Na-ion (aqueous)	Anode: NaTi_2_(PO_4_)_3_@C, Cathode: Activated Carbon	Na_2_SO_4_–SiO_2_ hydrogel with methanol additive	0.070 mS/cm at −30 °C	61.8 mAh/g at −30 °C	High stability at 0.13 A/g	—	—	—	Stable operation at −30 °C	[313]
Na-ion	Anode: metallic Na, Cathode: Na_2_Fe_2_(SO_4_)_3_	Hierarchical nanocellulose-based GPE	2.32 mS/cm	69.7 mAh/g after 50 cycles at 1C	Stable Na plating/stripping up to ±500 µA/cm^2^	—	—	—	Mesoporous structure ensures uniform ion flux and dendrite-free deposition	[314]
Na-ion (aqueous)	Anode: NaTi_2_(PO_4_)_3_, Cathode: Na_3_V_2_(PO_4_)_3_	Salt-concentrated methylated hydrogel	—	—	82.8% after 580 cycles	—	—	—	Suppressed water activity; stable cycling	[372]
Na-ion	Urchin-like MoS_2_/MoO_2_ microspheres coated with GO hydrogel	Na-ion compatible electrolyte (e.g., NaPF_6_ in EC/DEC)	Moderate to high (depending on GO dispersion)	~400–600 mAh/g (initial)	Excellent (>500 cycles with high retention)	Moderate (due to GO hydrogel)	Moderate	High (flexible hydrogel matrix)	High (GO hydrogel maintains integrity under strain)	[315]
Zn-ion	Na_3_V_2_(PO_4_)_3_@C@CNT porous network via hydrogel templating	Na-ion compatible (e.g., NaClO_4_ in EC/DEC)	High (due to CNT network)	~110–120 mAh/g (theoretical ~117 mAh/g)	Excellent (>1000 cycles with >90% retention)	High (CNT reinforcement)	High	Moderate to High	Very High (flexible and conductive network)	[312]
Zn-ion	Ultrathin Zn-based fiber electrodes	Solid-state gel or polymer electrolyte (e.g., PVA/ZnSO_4_)	Moderate to High (solid-state)	~100–150 mAh/g (depending on design)	Excellent (stable over hundreds of cycles)	High (fiber structure)	Moderate to High	High (textile-compatible)	Very High (maintains performance under bending/stretching)	[373]
Zn-ion	Zn-based electrodes with flexible current collectors	Hierarchical structured polymer electrolyte (e.g., PVA-based with Zn salts)	Moderate to High (solid-state polymer)	~100–140 mAh/g	Excellent (stable over 500+ cycles)	High (polymer matrix)	Moderate	High (wearable and flexible)	Very High (maintains capacity under bending/stretching)	[374]
Zn-ion	Zn-based electrodes with biopolymeric hydrogel coating	Aqueous ZnSO_4_ with hydrogel interphase	High (enhanced by hydrogel interface)	~120–160 mAh/g	Excellent (dendrite suppression enables long-term cycling)	Moderate to High (biopolymer matrix)	Moderate	High	Very High (stable under mechanical stress and deformation)	[324]
Al-ion	Mg-based electrodes (e.g., Mg foil or Mg alloy)	Modified aqueous electrolyte with antifreeze additives (e.g., Cl^−^/NO_3_^−^ coordination)	High (even at −50 °C)	~100–120 mAh/g	Excellent (stable at sub-zero temperatures)	Moderate	Moderate	Moderate	High (electrolyte remains functional under cold and stress)	[360]
Zn-ion	Hybrid Al-compatible electrodes (e.g., MnO_2_ cathode, Al anode)	Quasi-solid-state aqueous hybrid electrolyte (Al^3^⁺/H⁺ based)	High (optimized for dual-ion transport)	~250–300 mAh/g	Exceptional (10,000+ cycles with minimal degradation)	Moderate to High (gel matrix)	Moderate	High	Very High (smart switching maintains performance under stress)	[363]
Zn-ion	Fiber-shaped Al-compatible electrodes (e.g., MnO_2_ or conductive polymers)	Aqueous Al-ion electrolyte (e.g., AlCl_3_ or Al(NO_3_)_3_)	High (optimized for fiber geometry)	~200–300 mAh/g	Excellent (stable over many cycles under strain)	High (fiber structure)	Moderate to High	Very High (stretchable design)	Very High (maintains capacity under stretching and bending)	[364]
Zn-ion	Flexible Al-compatible electrodes (e.g., MnO_2_ cathode, Al anode)	Aqueous Al-ion electrolyte (e.g., Al(NO_3_)_3_ or AlCl_3_)	High (optimized for flexibility and ion transport)	~250–300 mAh/g	Excellent (long cycle life, >1000 cycles)	High (flexible substrate materials)	Moderate to High	High	Very High (maintains capacity under bending/stretching)	[365]
Zn-ion	Copper and zinc foil	Polyanion hydrogels: APTQ+/AAm	—	~160 mAh/g at 2 mA/cm^2^	—	50 kPa	—	~200%	—	[375]
Zn-ion	Anode: zinc, cathode: CoFe(CN)6	Zn(OTf)2	—	173.4 mAh/g at 0.3 A/g	No capacity decline after 2200 cycles	—	—	—	~100% under 180° bending	[376]
Zn-ion	Anode: zinc@graphite, cathode: α-MnO2	PAAm/ZnSO4/MnSO4	—	277.5 mAh/g at 1C	69.02% after 1000 cycles at 4C	—	—	—	>85% under 25% compression for 30 cycles	[377]

## 7. Challenges and Future Perspectives

### 7.1. Current Limitations in Hydrogel Applications

Hydrogels, owing to their unique properties, have garnered significant attention for a range of applications, including energy storage, sensors, and bioelectronics. However, there are several key limitations that restrict their broader integration in energy-related devices.

1. Mechanical Strength and Durability: Despite their impressive flexibility, many hydrogels face limitations in mechanical strength and durability, particularly when subjected to harsh operating conditions such as temperature extremes, high humidity, or mechanical deformation. This results in performance degradation, which limits their use in long-term applications like wearable electronics or energy storage systems [378].
2. Ionic Conductivity: The ionic conductivity of hydrogels, while suitable for some applications like supercapacitors, is often lower than that of traditional solid-state electrolytes or metal-based conductors. The challenge lies in optimizing the hydrogel matrix to improve ion transport without compromising other desirable properties such as biocompatibility and environmental.
3. Scalability and Manufacturing: The scalability of hydrogel-based devices, especially for large-scale energy storage applications, remains a significant hurdle. Many hydrogel-based systems are difficult to manufacture uniformly at a large scale while maintaining consistent performance.
4. Environmental and Biodegradability Concerns: While hydrogels are often considered eco-friendly, the degradation products of some synthetic hydrogels may raise concerns regarding their long-term environmental impact. Research is ongoing to develop fully biodegradable hydrogels that can break down harmlessly in natural environments.

### 7.2. Potential Solutions and Advancements

In addressing these limitations, several potential solutions and advancements are being explored:

1. Composite Hydrogels: The incorporation of conductive materials such as carbon nanotubes, graphene, and metallic nanoparticles into hydrogel matrices has shown promise in enhancing mechanical strength and conductivity. These composite hydrogels can offer the dual benefits of improved performance and flexibility, addressing the mechanical and conductivity issues simultaneously.
2. 3D Printing and Smart Fabrication Techniques: Advances in 3D printing and other smart fabrication methods are enabling the precise control of hydrogel structures, allowing for the creation of hydrogels with tailored properties for specific energy applications. This includes optimizing pore structures for ion transport or adjusting the polymer networks for improved mechanical integrity.
3. Self-Healing Hydrogels: Self-healing hydrogels, which can repair damage autonomously, offer an exciting avenue to overcome the durability challenges faced by hydrogels. By integrating dynamic covalent bonds or reversible crosslinking strategies, hydrogels can recover their function after being subjected to mechanical or environmental stress, which is crucial for ensuring long-term device reliability.
4. Biodegradable and Sustainable Hydrogels: Research into biodegradable and bio-based hydrogels, such as those derived from polysaccharides, is advancing rapidly. These hydrogels not only mitigate environmental concerns but also possess excellent biocompatibility, which is essential for energy devices that interact with the human body, such as wearable sensors and bioelectronics.

### 7.3. Future Research Directions

The future of hydrogel applications in energy materials and devices is promising, but several research directions need to be pursued:

1. Interdisciplinary Collaboration: There is a growing need for interdisciplinary research that combines materials science, chemistry, and engineering to create hydrogels with optimized properties for specific applications. Collaborations between researchers in fields such as nanotechnology, organic electronics, and biomaterials are key to developing next-generation hydrogels for energy storage and conversion systems.
2. High-Performance Batteries: Further research into the optimization of hydrogels for batteries, particularly for hybrid systems that combine both, is an exciting prospect. Focus should be on improving the energy density, stability, and cycling life of these devices through innovative hydrogel formulations.
3. Mechanistic Insights via Simulation: Although experimental studies have revealed the role of functional groups in facilitating ion transport within hydrogel electrolytes, deeper mechanistic insights are needed. Theoretical approaches such as density functional theory (DFT) and molecular dynamics (MD) simulations have been increasingly utilized to validate and explore ion–polymer interactions. DFT-based tight-binding methods can model electrochemical interfaces and predict ion–functional group interactions, while MD simulations reveal how polymer composition, crosslinking density, and hydration levels influence ion mobility and transport pathways [379,380]. Incorporating these computational tools in future studies will enhance the predictive design of hydrogel electrolytes and support the development of high-performance energy storage systems.

## 8. Conclusions

### 8.1. Summary of Key Points

Hydrogels represent a versatile and promising class of materials for energy storage and conversion applications. Their inherent properties—such as high water content, ionic conductivity, and mechanical adaptability—make them suitable for integration into batteries. Despite current challenges related to mechanical strength, scalability, and environmental impact, ongoing research into composite hydrogels, advanced fabrication techniques, and self-healing systems is paving the way for more robust and efficient devices. The future of hydrogel-based energy technologies lies in interdisciplinary collaboration and innovation in material design. As these materials continue to evolve, they are expected to play a critical role in shaping next-generation energy systems that are both sustainable and high-performing.

### 8.2. Impact of Hydrogels on the Future of Energy Materials and Devices

The integration of hydrogels into energy devices has the potential to revolutionize the energy sector. Hydrogels, particularly when optimized for high-performance applications, could lead to the development of more sustainable, flexible, and environmentally friendly energy storage systems. Their inherent properties, such as high-water content, ionic conductivity, and biocompatibility, make them ideal candidates for next-generation energy devices, including wearable electronics, flexible sensors, and efficient energy storage units.

### 8.3. Final Thoughts

As the field of hydrogel-based energy materials continues to evolve, the future holds great promise. Advances in material design, fabrication techniques, and functionalization strategies are expected to overcome current limitations, leading to new applications in a variety of fields, from wearable devices to large-scale energy storage systems. By addressing the challenges and pushing the boundaries of hydrogel research, these materials could play a crucial role in the development of next-generation energy systems special rechargable batteries that are both efficient and sustainable.

## Data Availability

No new data were created or analyzed in this study.
